# Estimating the Population Health Impact of Recently Introduced Modified Risk Tobacco Products: A Comparison of Different Approaches

**DOI:** 10.1093/ntr/ntaa102

**Published:** 2020-06-04

**Authors:** Peter N Lee, David Abrams, Annette Bachand, Gizelle Baker, Ryan Black, Oscar Camacho, Geoffrey Curtin, Smilja Djurdjevic, Andrew Hill, David Mendez, Raheema S Muhammad-Kah, Jose Luis Murillo, Raymond Niaura, Yezdi B Pithawalla, Bill Poland, Sandra Sulsky, Lai Wei, Rolf Weitkunat

**Affiliations:** 1 Medical Statistics and Epidemiology, P N Lee Statistics and Computing Ltd, Sutton, Surrey, UK; 2 Social and Behavioral Sciences, NYU School of Public Health, New York, NY; 3 Health Sciences, Ramboll US Corporation, Amherst, MA; 4 Clinical Science and Epidemiology, Philip Morris R&D, Philip Morris Products SA, Neuchâtel, Switzerland; 5 Regulatory Affairs, Altria Client Services LLC, Richmond, VA; 6 Computational Tools and Statistics, British American Tobacco (Investments) Ltd, Group R&D, Southampton, UK; 7 Scientific and Regulatory Affairs, Reynolds American Inc Services Company, Winston-Salem, NC; 8 Modelling, Ventana Systems UK Ltd, Salisbury, UK; 9 Department of Health Management and Policy School of Public Health, University of Michigan, Ann Arbor, MI; 10 Strategic Consulting, Certara USA Inc, Menlo Park, CA

## Abstract

**Introduction:**

Various approaches have been used to estimate the population health impact of introducing a Modified Risk Tobacco Product (MRTP).

**Aims and Methods:**

We aimed to compare and contrast aspects of models considering effects on mortality that were known to experts attending a meeting on models in 2018.

**Results:**

Thirteen models are described, some focussing on e-cigarettes, others more general. Most models are cohort-based, comparing results with or without MRTP introduction. They typically start with a population with known smoking habits and then use transition probabilities either to update smoking habits in the “null scenario” or joint smoking and MRTP habits in an “alternative scenario”. The models vary in the tobacco groups and transition probabilities considered. Based on aspects of the tobacco history developed, the models compare mortality risks, and sometimes life-years lost and health costs, between scenarios. Estimating effects on population health depends on frequency of use of the MRTP and smoking, and the extent to which the products expose users to harmful constituents. Strengths and weaknesses of the approaches are summarized.

**Conclusions:**

Despite methodological differences, most modellers have assumed the increase in risk of mortality from MRTP use, relative to that from cigarette smoking, to be very low and have concluded that MRTP introduction is likely to have a beneficial impact. Further model development, supplemented by preliminary results from well-designed epidemiological studies, should enable more precise prediction of the anticipated effects of MRTP introduction.

**Implications:**

There is a need to estimate the population health impact of introducing modified risk nicotine-containing products for smokers unwilling or unable to quit. This paper reviews a variety of modeling methodologies proposed to do this, and discusses the implications of the different approaches. It should assist modelers in refining and improving their models, and help toward providing authorities with more reliable estimates.

## Introduction

Smoking-related mortality is a major public health issue. Harm reduction strategies include encouraging smokers to quit, and introducing modified risk nicotine-containing products for smokers unwilling or unable to quit. Recent new products include tobacco heated products, smokeless tobacco, and e-cigarettes. The US FDA guidance on completing modified risk tobacco product (MRTP) applications^1^ includes estimating population-level health effects of introducing MRTPs. Consequently, various publications have described modeling approaches to assess the impact on mortality of introducing MRTPs.

In this review, we ignore models of effects of MRTP introduction on tobacco use^2,3^ not considering mortality, models of effects of tobacco control strategies on other health measures (eg, ^4–13^), and true agent-based models,^14^ where one individual’s action can affect another. We also ignore two models with limited utility due to strong simplifying assumptions.^15,16^

By mid-2018 we knew of two relevant unpublished and 11 published models,^17–35^ six supported by tobacco companies and seven by public funding (see Table 1). Most of us attended a meeting on models at New York University in 2018, and the paper is written by those who attended and or authored the papers described above. The 13 approaches are referred to subsequently by the model abbreviations shown in Table 1. Publications on 11 models were previously available at the New York meeting. ALCS1 and ALCS2 were described at the meeting^36^ as the cohort and agent-based models, full details of ALCS1 being published later.^37^

**Table 1.** The 13 Models—Publications and Support

Model	Supported by	Authors and publications
Tobacco companies		
ALCS1	Altria Client Services	Black et al. (2018)^36^ and Muhammad-Kah et al. (2019)^37^
ALCS2	Altria Client Services	Black et al. (2018)^36^
BAT	British-American Tobacco	Hill and Camacho (2017)^17^
JTI	Japan Tobacco International	Poland and Teischinger (2017)^18^
PMI	Philip Morris International	Weitkunat et al. (2015)^21^ and Djurdjevic et al. (2018)^23,24^
RJR	Reynolds American Inc Services Company	Bachand and Sulsky (2013)^19^ and Bachand et al. (2018)^20^
Public funding		
CTDRP	California Tobacco-Related Disease Research Program	Tengs et al. (2001, 2004, 2005)^25–28^
FDA	US Food and Drug Administration	Vugrin et al. (2015)^29^ and Apelberg et al. (2018)^30^
KALK	National Institutes of Health, National Cancer Institute, US Food and Drug Administration	Kalkhoran and Glantz (2015)^33^
LEVY1	National Institute on Drug Abuse, Cancer Intervention and Surveillance Modeling Network, National Cancer Institute	Levy et al. (2017)^34^
LEVY2	National Institute on Drug Abuse, Cancer Intervention and Surveillance Modeling Network, National Cancer Institute	Levy et al. (2018)^35^
NIH	National Institutes of Health	Soneji et al. (2018)^31^
UM	University of Michigan	Warner and Mendez (2019)^32^

Of the 13 models, 10 are cohort-based, comparing a null scenario, where MRTPs are not introduced, and alternative scenarios, where they are. These are considered first in some detail. The other three models (CTDRP, KALK, and NIH) are essentially different, and are described separately later. When developing a cohort model, a variety of choices has to be made, which are summarized in Table 2. Our objective is not to decide which model is the best, partly as formal validation of the models is not possible. The models have different strengths and weaknesses. Rather, we aim to compare and contrast the models, give insight into their nature, comment on the differences in approach, and recommend possible modeling extensions. We do not describe each approach fully, although more information is given in the Supplementary Files. Supplementary File 1 includes five tables: Tables 1 and 2 summarize 48 model characteristics separately for the six tobacco-industry sponsored and the four publicly funded models. Tables 3 and 4 summarize the differing sets of tobacco transition probabilities (TTPs) used to develop tobacco histories in the null and the alternative scenarios. Table 5 gives fuller mathematical details of how the estimates of population health impact were derived from the tobacco histories. Even fuller details are given in the source publications.

**Table 2.** Considerations in the Choice of a Cohort Model

Scope	Type(s) of new product to be evaluated
	Location for which estimates are relevant
	Age groups and sexes for which results required
	Health endpoints to be considered; mortality, loss of life, and quality of life
	Whether results for specific diseases required
Initial population	Year of baseline
	Smoking groups used at baseline
	Age at baseline
Follow-up	Year of end of follow-up
	Add new young individuals and immigrants during follow-up?
	Remove deaths and emigrants during follow-up?
	Follow individuals or groups?
	Tobacco groups to be used in the alternative scenario
	In the null scenario are initiation, cessation, and re-initiation all to be considered?
	In the alternative scenario which of the possible initiations, cessations, re-initiations, and switches are to be considered?
	Can these transition probabilities vary by period of follow-up?
	Can they vary by previous smoking habits?
Estimating health endpoints from smoking histories	What *F*-factor is to be assumed for estimating the relative increase in risk for MRTP users and smokers?
	What *G*-factor is to be assumed for estimating the relative increase in risk for dual users and smokers?
	Allow for other factors such as environmental tobacco smoke?
	How is risk for a given smoking history, relative to never exposed individuals and groups, to be determined?
Sources of data used	These include initial population distributions by sex, age, and smoking, immigration, emigration and birth rates, death rates, transition probabilities, *F*- and *G*-factors, and current and former smoking relative risks

MRTP = modified risk tobacco product.

## Comparisons of the Cohort-Based Models

### Scope of the Models

The publications concern e-cigarettes (or vaporized nicotine-containing products [VNPs]) for four models (ALCS2, LEVY1, LEVY2, and UM). Others are more general, using terms such as MRTP (ALCS1, JTI, PMI, and RJR), new nicotine product (BAT), or new tobacco product (FDA). Except for BAT which reported UK results, all other models reported results only for the United States. However, these approaches are applicable to any MRTP or country, given suitable input data.

Most models concern mortality in at least most of the adult population, seven (ALCS1, ALCS2, FDA, LEVY1, LEVY2, PMI, and RJR) presenting results by age and three (BAT, JTI, and UM) not doing so. All models use age-specific inputs, and in principle could make age-specific predictions.

Most models presented all-cause mortality results. PMI limited attention to lung cancer, chronic obstructive pulmonary disease, heart disease, and stroke, which contribute about two-thirds of smoking-related mortality, their methodology requiring disease-specific estimates of the relative risk (compared with never-smokers) in current and former smokers, reliable evidence being unavailable for all smoking-related diseases. Most models only estimated death counts and or rates (ALCS2, BAT, FDA, JTI, and LEVY2), though some additionally presented results for life-years lost (ALCS1, LEVY1, PMI, RJR, and UM). RJR also considers quality of life. Deciding whether MRTP introduction is beneficial seems unlikely to depend on the endpoint.

Results were given by gender for five models (ALCS2, FDA, LEVY1, PMI, and RJR), while one (UM) has not published gender-specific results. ALCS1, BAT, JTI, and LEVY2 reported results only for genders combined or a single gender, using gender-dependent data. Especially in countries where smoking prevalence varies markedly by gender, gender-specific calculations seem preferable. While information by gender on the likely uptake of some MRTPs may currently be limited, sensitivity analyses can investigate different assumptions about uptake.

### Types of Model Used

The typical scenario in the remaining 10 models is a cohort-based approach, following a population over time under null and alternative scenarios. At intervals during follow-up the distribution of tobacco habits for each scenario may change, rates of transition between tobacco groups being governed by TTPs. Coupled with estimates of the increase in risk of disease for exclusive and dual use of the MRTP, expressed relative to the increase in risk from exclusive smoking, the information built up on patterns of tobacco use over time in the two scenarios is then used to estimate the mortality change associated with MRTP introduction.

In the sections following we consider various aspects of the modeling, highlighting between-model differences in their characteristics. Fuller details are given in the Supplementary Material.

### Initial Population and Follow-up

Various names (eg, status quo, base, and core) are used to describe the no-MRTP situation—the null scenario. Various names (eg, modified base case, NGP, counterfactual) also describe the MRTP situation—the alternative scenario. RJR can also have a two-product null scenario, with a third being introduced in the alternative. ALCS1 allows comparison of a two-product null scenario with a two-product alternative. Here the second product is assumed already on the market, the alternative scenario allowing estimation of the effect of introducing a modified risk claim on it.

Three models start with a population of a specified age (RJR 12, ALCS1 13, and LEVY1 15 years), and two with a specified age range (PMI 10–79 and LEVY2 15–99 years). None of these increments the population during follow-up, either with new young individuals or immigrants. ALCS1 and RJR remove deaths during follow-up, but PMI do not, though describing a correction for differential survival. LEVY1 and LEVY2 do not remove deaths. We describe these models as using a “single cohort” approach.

Three models (ALCS2, BAT, and FDA) start with an all age population, adding births and immigrations, and removing deaths and emigrations. Two (JTI and UM) are similar, but only consider a population aged 18+ years, adding new 18-year olds, and removing deaths, but ignoring immigration or emigration. We describe these models as using a “multiple cohort” approach.

Some models (ALCS2, JTI, and PMI) can be called “microsimulation” models, in which each individual is separately followed over time using pseudo-random numbers to determine transitions between product states. Others (BAT, FDA, LEVY1, LEVY2, and UM) consider groups not individuals, not using random numbers. RJR and ALCS1 consider groups but takes uncertainty in the input data into account, providing uncertainty estimates around outcome values.

Most reported applications concern follow-up from a recent year to the distant future. The periods studied from MRTP introduction range from 88 years (2012–2100) for JTI, 84 (2016–2100) for LEVY2 and one FDA model application,^30^ 71 for LEVY1 (2012–2083), 60 for ALCS1 (2015–2075), ALCS2 (2000–2060), and UM (2010–2070), and 50 (2000–2050) for BAT and the original FDA publication.^29^ RJR uses life table techniques to follow 12-year-old never tobacco users until all die. Exceptionally, PMI uses a “hindcasting” approach, considering a past period (1990–2010). Doubtless, all the models could be implemented using different periods.

None of these approaches have major weaknesses. PMI’s hindcasting approach avoids making inferences about events many years hence, although the short follow-up means hypothetical effects of the MRTP being a gateway to cigarettes are hardly considered, such initiators not becoming old enough to be at major disease risk. Consideration of immigration or emigration seems unimportant, as it applies to both the null and alternative scenarios.

Using a single or multiple cohort approach seems a matter of choice. While the latter allows inferences about the whole population during follow-up, a single cohort approach provides information similar to that from an epidemiological cohort study, often used to study effects of smoking. One can use multiple single cohorts with different exposure histories to more fully determine population mortality effects, as demonstrated using the ALCS1 and RJR models.

That some single cohort approaches do not remove deaths may be more relevant. PMI showed^22^ that adjusting for differential mortality between scenarios had little effect, but this may not apply for follow-up periods longer than their 20 years.

### Specifying the Initial Population

The approaches following birth cohorts from a specified young age (ALCS1, LEVY1, and RJR) assume they are initially never-smokers. The other seven, starting with a broad age range, subdivide the initial population into never, current, or former smokers. All but one (LEVY2) further separate former smokers by time quit. Only JTI subdivides ever smokers by amount smoked. In all these seven approaches smoking habit distributions derive from national data for the start year.

### Tobacco Transition Probabilities

During follow-up, TTPs determine whether an individual will switch tobacco group in a given period. The period is typically a year, though PMI also allows tobacco habits to change every month or 3 months.

In all models, the null scenario represents the status quo, the commonest being one with three smoking groups (never, current, and former) and three transitions (initiation, cessation, and re-initiation). Sometimes the null involves two products (eg, cigarettes and another product). See also Figure 1 in Supplementary File 2.

Table 3 of Supplementary File 1 shows that most models allow three transitions, though JTI, LEVY1, and UM only allow initiation in a restricted age period, and some disallow re-initiation (JTI, LEVY2, and UM) or only allow it once (RJR). Models disallowing re-initiation use the “established cessation rates” of CISNET researchers^38^ on smokers quitting for at least 2 years. Except for LEVY1 and UM, all models allow variation in TTPs by age and gender, and six (ALCS1, ALCS2, BAT, FDA, PMI, and RJR) allow variation by aspects of smoking or length of follow-up.

In the alternative scenario, the number of groups varies by model. Three (JTI, LEVY1, and PMI) consider five (never either product, current cigarette-only, current MRTP-only, current dual use, and former product use) with those currently using one product and formerly the other classified by current use. Two models (ALCS1 and BAT) consider seven groups, subdividing former tobacco users into previous cigarette-only, MRTP-only, or dual use. ALCS2, FDA, and RJR extend the ALCS1 and BAT groups by subdividing current cigarette-only and MRTP-only by never and former use of the other product, producing nine groups. LEVY2 considers five groups, the seven of ALCS1, BAT, and RJR except current and former dual use. UM only considers cigarette initiation and cessation, using three groups. See also Figure 2 in Supplementary File 2 which shows the transitions in the PMI model.

Table 4 of Supplementary File 1 summarizes the TTPs in the alternative scenario. Five models markedly differ. LEVY1 subdivides never-smokers initially by whether they would (A) or would not (B) have started smoking in the absence of e-cigarettes, with A and B further subdivided by whether they try (1) or not try (2) VNPs. A1 and B1 are then subdivided by whether they try, then quit e-cigarettes, or continue to use them. Each subgroup ends up as long-term users of never tobacco, single product, or dual use. With long-term use, no further transitions occur. LEVY2, concerned with replacing cigarettes with e-cigarettes, allows initiation only with e-cigarettes in the alternative model, quitting rates being as in the null scenario. Re-initiation and switching are irrelevant. In the alternative scenario, RJR, ALCS1, and FDA subdivide never-smokers initially by whether they would have initiated smoking, and current smokers by whether they would have quit. Different transition probabilities for MRTP initiation and switching to MRTP use (and dual use) can be entered according to these subdivisions.

BAT, FDA, and PMI consider all possible TTPs for initiation, quitting, re-initiation, and switching, given the tobacco groups considered, while ALCS1, ALCS2, and RJR disallow initiation or re-initiation to dual use, requiring two successive periods for this. RJR also disallows transitions from dual to single product use, dual users only quitting both or remaining dual users. The JTI model diagram in Supplementary Figure S1 of the source^18^ disallows initiation by never users (only by the new incoming population), or complete quitting of tobacco use in one transition for dual users, and only allows re-initiation to MRTP.

In the microsimulation approaches, variability in the estimated population health impact can be assessed by carrying out different software runs with different random numbers. In two approaches not using random numbers, RJR and ALCS1 also allow addressing of uncertainty by carrying out different software runs where the TTP values are randomly drawn from a truncated normal distribution.

In the null scenario, all models use national data as their source for TTPs, often using initiation and cessation rates from CISNET.^38^ In the two-product null scenario, ALCS1 derives transitions relating to the second product (smokeless tobacco) from published data.^39^ For their one and two-product nulls, RJR uses best available data from national estimates and consumer testing. TTPs can be entered as fixed or random. Some models (ALCS2, BAT, FDA, JTI, and PMI) validate or calibrate rates by comparing observed and predicted smoking prevalence some years from baseline. For validation, RJR and ALCS1 predict current life tables using past input data.

The RJR validation use US data for the null scenario and Swedish data for the alternative, ALCS1 using US life tables (single cohort) and US Census Bureau data (multiple cohort). ALCS2, BAT, and FDA also compare predicted prevalences with other projections. ALCS2, FDA, and JTI validate by comparing modeled US population and mortality projections with US Census Bureau estimates.

Alternative scenario TTPs are usually hypothetical, illustrating different situations. However, ALCS1 estimates TTPs from a study in which participants were asked about their behavioral intentions and intent to purchase, while ALCS2 uses recent data on smoking and e-cigarette use from the Population Assessment of Tobacco and Health study. LEVY1 develops TTP estimates for their VNP scenario “from recent literature.” RJR uses data from consumer intention studies as starting points for sensitivity analyses.

While apparently preferable to use many tobacco groups in the alternative scenario and allow for all possible transitions, note that the difficulty of generating plausible TTPs increases with model flexibility, particularly if groups are subdivided by amount used.

Note also that if, for a given year, a model estimates the risk from tobacco history (relative to the unexposed) based only on that estimated at the previous year and the individual’s tobacco use in the latest year, it is unnecessary to have data available at each year on the products previously smoked. The estimated risk will still depend on previous history.

### Risk Assumed for MRTP-Only Users and Dual Users Compared With Cigarette-Only Smokers

UM, which only considers effects of e-cigarette introduction on cigarette initiation and cessation rates, assigns the same risk increase to dual users and smokers. In their null scenario, e-cigarettes are considered harmless. However, their sensitivity analyses assume each smoker quitting smoking following e-cigarette use loses 10% of the mortality reduction associated with direct quitting. Other models use the risk increase from exclusive MRTP use relative to that from exclusive smoking, which PMI call an *F*-factor.

In their main analyses the *F*-factor is assumed no greater than 0.10 in seven models (JTI 0.04–0.10; ALCS2, BAT, LEVY1, LEVY2 0.05; RJR 0.08; ALCS1 0.09), the low values partly reflecting the opinions of experts assembled to rank twelve nicotine-containing products by harm.^40,41^ PMI and FDA use higher values, 0.20 and 0.25, based on toxicology and clinical studies. The chosen *F*-factor may reflect the MRTP to be introduced. The *F*-factor for ALCS1, for smokeless tobacco, was derived by analyzing National Health Interview Survey data linked to National Death Index data. Some models reflect uncertainty by giving results for varying *F*-factors. Exceptionally, RJR incorporates uncertainty directly, allowing the *F*-factor to be drawn from a truncated normal distribution, the mean being used as the best estimate.

The models also vary concerning the *G*-factor, the increase in risk of dual users relative to that in exclusive smokers. Ignoring LEVY2, which ignores dual use, five models (ALCS1, ALCS2, BAT, FDA, and RJR) assume dual users have identical risk to cigarette-only smokers (*G* = 1), but three assume a lower value. JTI assumes dual users have the combined excess risks for exclusive use of each product (1 + *F*), but account for an estimated 42% reduction in cigarette consumption in dual users. LEVY1 assumes a *G*-factor of 0.70, but consider alternatives from 0.50 to 1.00. Assuming dual users reduce cigarette consumption by 50%, PMI set *G* = (1+ *F*)/2 in many sensitivity analyses, but also provide results for *G* from 0.4 to 2.0.

Without epidemiological data, most MRTPs being quite new, considerable uncertainty must exist about the correct *F*- and *G*-factors. *F*-factors for a product can initially only be obtained from data on biomarkers and short-term clinical endpoints. The *G*-factor also requires reliable data on cigarette consumption differences between exclusive smokers and dual users.

### Approaches for Estimating Risks Relative to Never-Smokers

For each model considered below, fuller details are available, not only in the source papers but also in Table 5 of Supplementary File 1.

For each scenario, PMI estimates an individual’s risk, relative to that of a never tobacco user, by disease and time of follow-up using an extension of the negative exponential model (NEM)^22^ which considers the full tobacco history. A full description of the extended NEM, justification for its use, and examples of its application are available.^24,42^ It requires knowledge of exposure at each time, and also disease- and age-specific estimates of the current cigarette smoking relative risk and the quitting half-life, the time a quitter takes to halve the excess risk from continued smoking. These estimates derive from published meta-analyses.^22^ JTI uses the same approach, but differing relative risks (for overall, not cause-specific mortality),^43^ and decay curves following quitting.^18^

BAT assumes mortality rates depend on age, gender, and smoking and in former smokers decline with time quit according to a NEM. However, not all aspects of smoking history are considered. Notably, worst-case assumptions are made that quitters who relapse have the risk of smokers at the age they relapse, and that late and early starting smokers have the same risk. They use UK-based relative risk estimates.^17^

RJR relates mortality rates (by gender) to age, years smoked, and years using a Poisson model based on Kaiser Permanente Study data^44^ and US Census data. The model coefficients are estimated using a Bayesian approach, allowing for calculation of uncertainty intervals for the modeled survival estimates. In their main analyses, current and former MRTP-only users are assumed to have 8% (or 11%) of the increase in risk of current and former smokers. For switchers between products, mortality rates are derived by multiplying factors representing background rates for the age range used, and duration of current and former use of each product.

ALCS1 uses a similar procedure except three separate product-specific all-cause mortality models are used for never tobacco users, cigarette smokers, and former cigarette smokers.

The ALCS2 models the gender-specific probability of all-cause mortality using the Gompertz equation and is based on gender-specific mortality data from the Kaiser Permanente Study which is segregated by age, years smoked, and years quit. These rates were adjusted to the 2000 US population using Human Mortality Database tables. Mortality rates are also adjusted over the 60-year period using Lee–Carter mortality improvement factors.^45,46^

FDA derives risk estimates by gender, age, smoking status, and age quit from National Health Interview Study data. Current and former MRTP-only users are assumed to have a risk a fixed proportion of that for current and former smokers, while current dual users are assigned the maximum of the individual risks, as is also true for former dual users. Current smokers formerly using MRTP are assigned the risk of current smokers, while former smokers currently using MRTP are assigned a risk (1 − *F*) times that for former smokers plus *F* times that for current smokers.

UM derives relative risk estimates by gender, age, smoking status, and years quit from Cancer Prevention Study II (CPS II) data, though most analyses collapse estimates over gender.

LEVY1 determines risks dependent only on the proportions classified as never tobacco users, or long-term smokers, MRTP-only smokers, or dual users. Subsequent quitting or switching is ignored, so only risks from current use are relevant. For current smokers, the age- and gender-dependent relative risks come from CPS II and the earlier Cancer Prevention Study I (CPS I) study, while for MRTP-only smokers or dual users, relative risks are derived using the *F*- and *G*-factors described earlier.

LEVY2, which only considers replacement of cigarettes by e-cigarettes, again derives age- and gender-dependent relative risks from CPS I and II, but for former and current smokers. Risk of current MRTP-only users is determined using a defined *F*-factor, while former smokers switching to MRTP have an increase in risk (1 − *F*) times that for former smokers plus *F* times that for current smokers. Dual users and RTP users switching to cigarettes are not considered.

Similarities and differences exist between approaches. The extended NEM allows risk estimation for complex changes in exposure. While it fits well the decline in risk following quitting for various diseases^47–50^ and evidence exists that it fits the decline following dose-reduction for lung cancer,^42^ its validity is untested for more complex patterns of change. The RJR model relates risk to age, duration of exposure and time quit. While not fully describing patterns of risk, their approach successfully predicted current US and Swedish life tables using historical input data.

Models relating risk to duration and time quit may incompletely describe risk patterns. Thus, risk may differ between someone smoking 20 cigarettes per day at ages 21–40 and 10 at 41–60, and someone with the reverse pattern, or between two smokers of the same age with the same duration, but smoking at different ages. Without a generally accepted, validated model to estimate risk for complex patterns of changes in smoking, can one achieve a validated model for more complex patterns involving smoking and MRTP use?

Approaches differ on how to estimate relative risks for smokers compared with never tobacco users. Whereas PMI derives relative risks (and half-lives) from published meta-analyses of epidemiological data,^22^ other models use estimates from specific studies, some generalizing US Census estimates. Given the differences between models in the smoking relative risks used, comparisons between models of changes in mortality from MRTP introduction may be more meaningfully expressed in proportional rather than absolute terms. If, for a model, numbers of smoking-related deaths in the null and alternative scenarios are estimated as *X* and *Y*, the reduction in deaths from MRTP introduction may be better expressed as (*X* − *Y*)/*X*, rather than *X* − *Y*, given both *X* and *Y* depend on the assumed relative risk for smoking.

Alternatively, RJR focuses on “tipping point analyses” which estimate the proportion of the population that must engage in a beneficial exposure shift to counterbalance the effect of any harmful transitions.

### Estimating Mortality Attributable to Smoking

For each follow-up year and age group, PMI uses national age- and gender-specific estimates of population and deaths by cause, together with the mean relative risk estimated as described earlier, to estimate death rates in never tobacco users, and hence numbers and rates of death attributable to smoking in each scenario, differences between scenario quantifying the effect of MRTP introduction. This approach is facilitated by PMI typically considering a follow-up period of 1990–2010, where national rates are available throughout, so allowing for changing never-smoker rates over time.

Other approaches typically project into the future, and assume never-smoker rates are invariant. While the models do not predict changes in mortality associated with factors other than smoking, never-smoker rates may change for various reasons, including advances in medical science. This emphasizes why changes in mortality from MRTP introduction are better presented as a proportion (or through tipping point analyses), rather than in absolute terms.

### Conclusions From the Cohort-Based Approaches

Table 3 summarizes the conclusions reported from the 10 approaches. Despite differences in inputs and methodology, these generally agree that introducing an MRTP very likely has a beneficial health impact. While FDA, in their original paper describing their methodology^29,30^ suggests potential benefits from smokers switching to the MRTP could be offset by increased initiation, others (PMI, RJR, and UM) suggest a net benefit is still more likely than not. One cannot draw completely general conclusions—thus MRTP introduction into a population not smoking cigarettes can hardly be beneficial, assuming the MRTP retains some of the mortality risk of cigarettes.

**Table 3.** Some Conclusions From the Cohort-Based Approaches

Approach	Reference	Conclusion
ALCS1	^37^	“Our sensitivity analyses using various reasonable ranges of input parameters do not indicate any scenario under which the net benefit [of introducing a modified risk claim on an existing smokeless tobacco product] could be offset entirely.”
ALCS2	^36^	“Sensitivity analysis results demonstrate that, under defined conditions, relatively large changes to the ERR of e-cigarettes [i.e. increases in the F-factor to 0.4] would still result in a net benefit to the population].”
BAT	^17^	“The results suggested an overall beneficial effect from launching e-cigarettes and that system dynamics could be a useful approach to assess the potential population health effects of nicotine products when epidemiological data are not available.”
JTI	^18^	“Models to project population effects of an MRTP should account for possible mortality effects of reduced smoking among dual users.”
PMI	^22^	“The mortality reduction is proportional to the dose reduction, increasing rapidly with time of follow-up. Plausible increases in re-initiation or dual users’ consumption, or decreased quitting by smokers would not eliminate the drop.”
RJR	^20^	“… within a single birth cohort, switching completely from cigarette smoking to MRTP use is more likely to lead to a population-level survival benefit than initiating tobacco use with an MRTP instead of cigarettes.”
FDA	^29^	“Potential benefits from cigarette smokers switching to the lower-risk product can be offset over time through increased initiation of this product.”
FDA	^30^	“Enacting a regulation to lower the nicotine content of cigarettes to minimally addictive levels in the United States would lead to a substantial reduction in tobacco-related mortality, despite uncertainty about the precise magnitude of the effects on smoking behaviors.”
LEVY1	^34^	“Under most plausible scenarios, VNP use generally has a positive public health impact. However, very high VNP use rates could result in net harms.”
LEVY2	^35^	“Our projections show that a strategy of replacing cigarette smoking with vaping would yield substantial life year gains, even under pessimistic assumptions regarding cessation, initiation and relative harm.”
UM	^32^	“Our analysis strongly suggests that the upside health benefit associated with e-cigarettes, in terms of their potential to increase adult smoking cessation, exceeds their downside risk to health as a result of their possibly increasing the number of youthful smoking initiators.”

The six tobacco industry sponsored models are shown first. MRTP = modified risk tobacco product.

Supplementary File 1 provides information on the population health impact estimated by the 10 approaches. Due to differences between models in factors such as length of follow-up period, the diseases, sexes, health endpoints considered, and the assumed values of the *F*- and *G*-factor, it is not straightforward to compare these estimates.

Strengths and weaknesses of the 10 approaches are considered in the discussion.

## The Other Three Models

### The CTDRP Model

Whereas alternative scenarios in cohort-based approaches typically start with never tobacco users with a known distribution of smoking, which then converts over time to a combined distribution of smoking and MRTP use, the CTDRP model only considers the distribution of cigarette smoking. The model, described in 2001,^25,26^ was later used to study the health impact of introducing policies to mandate either safer cigarettes^27^ or nicotine reduction in cigarettes.^28^

Both applications consider 50-year follow-up from 2003. In the safer cigarette application, cigarettes initially available are immediately replaced by safer cigarettes, while in the nicotine reduction application, the new cigarettes become the only ones available in 2009. In both applications, existing rates of initiation, cessation, and re-initiation without introduction of the new cigarette are modified according to various assumptions, with the effect this has on quality-adjusted life-years quantified.

Compared with the mortality difference between unmodified cigarette smokers and never-smokers, modified cigarette smokers have a difference reduced by various percentages. However, the authors recognize that the improvement in survival prospects of current smokers would be proportional to the time they smoked modified versus unmodified cigarettes. Surprisingly, since smoking starts after birth, age is taken as a proxy for time. Thus, for example, a smoker aged 40 at the time the switch to the modified cigarettes occurred, would, at age 60, have a mortality reduction only one-third (20/60) of that for lifetime use. Similar methods estimate reductions in mortality of former smokers. In the nicotine reduction application an adjustment is made for smokers compensating by increasing consumption or modifying manner of smoking. That model also accounts for an assumed percentage of smokers entering the black market to purchase unmodified cigarettes. In the safer cigarettes application, the modeling also adjusts for those choosing to smoke them having lower morbidity rates as well as improved survival, so the reduction in quality of life associated with smoking would be by an amount consistent with the mortality reduction.

The safer cigarettes publication^27^ concludes that “Our simulation results reveal that even if requiring cigarettes to be safer makes smoking more attractive and increases tobacco use, a net gain in population health is still possible,” while that on nicotine-reduced cigarettes^28^ concludes that “Despite any mortality increases due to compensatory smoking or the emergence of a black market, implementation of the AMA proposal would likely prevent the addiction of scores of new smokers and result in important gains to the nation’s health.” (AMA = American Medical Association).

The Tobacco Policy Model is interesting, but concerns a situation essentially different from the cohort-based models, where in all alternative scenarios, the two products are assumed available simultaneously.

### The KALK Model

Whereas the cohort-based approaches follow a population through time (under both scenarios) to determine tobacco habit changes, KALK estimates the distribution of habits in the two scenarios in 2013. In the null scenario, initiation rates of cigarette smoking and e-cigarette use are specified, with cigarette initiators subdivided by interest in quitting. Subsequently, those uninterested in quitting are subdivided into those remaining cigarette smokers and those trying e-cigarettes, while those interested are divided into those who try e-cigarettes, try other methods, try unassisted, or make no quit attempt. Those initiating with e-cigarettes, or trying them after cigarettes, may end up as quitters, users of one product, or dual users, while those who do not do so end up as quitters or cigarette smokers. Seven different alternative scenarios modify these initial populations. Subsequently, health costs are assigned on the scale never tobacco 0 units, current cigarette-only 100, current dual users 95, and former users 10, with current e-cigarette users assigned values from 1 to 50.

The authors conclude “widespread promotion of e-cigarettes may have a wide range of population-level health effects; depending on both e-cigarette health risks and patterns of use,” and that the varying health effects “suggest a potential for harm.” There are various disadvantages compared with the cohort-based approaches. Firstly, health costs depend on final tobacco status, which cannot change during life. Also, age is not considered. Furthermore, it is unclear why health care costs of former users are only 10% of those for current cigarette smokers, as it takes many years quitting to achieve such a reduction in the increase in risk of disease from smoking, about 30 years for lung cancer and chronic obstructive pulmonary disease.^48,50^ To assume, as in some alternative scenarios, that e-cigarettes have an *F*-factor half that for cigarettes is inconsistent with the lower estimates generally assumed. Others note that KALK’s pessimistic estimates also apparently result from very high rates of smoking initiation following e-cigarette use.^34^ It is also unclear why cigarette initiators interested in quitting, have quit rates lower for those attempting quitting using e-cigarettes than for those attempting it using nicotine replacement therapy or medication.

### The NIH Model

This approach has two main parts.

The first concerns the estimated 3 490 000 current cigarette smokers aged 25–69 years who, in 2014, had attempted to quit smoking in the past year, and also currently used e-cigarettes. The authors estimate the increases from using e-cigarettes as a cessation tool, in the number quitting and remaining abstinent for at least 7 years, and in years of life gained.

The second concerns the estimated 3 640 000 who in 2014 were 12–29-year-old never-smokers, the authors estimating years of life lost due to the increase in those eventually becoming current daily cigarette smokers at age 35–39 through e-cigarette use. The authors estimate that, due to e-cigarette use, 2070 additional current smoking adults would quit, and 168 000 additional young never-smokers would initiate, leading to 1 510 000 years of life lost (95% confidence interval 920 000–2 160 000). These estimates derive from assuming e-cigarette users had a 95% harm reduction compared with smokers. The years of life lost increased to 1 600 000 for a 50% reduction.

The authors conclude that “based on the existing scientific evidence related to e-cigarettes and optimistic assumptions about the relative harm of e-cigarette use compared to cigarette smoking, e-cigarette use currently represents more population harm than benefit.”

The approach has various weaknesses, including assuming that only those attempting quitting in the last year will switch to e-cigarettes and that the probability of re-initiating smoking is independent of e-cigarette use.

Also, for adults, it uses an estimate of 0.86 (95% confidence interval 0.60–1.23) associated with e-cigarette use for the odds ratio of quitting among smokers interested in quitting. This estimate^51^ was criticized by experts^52^ who consider introducing e-cigarettes increases the number of quitters.

In young people, their adjusted odds ratio estimate for cigarette smoking initiation for ever versus never e-cigarette users of 3.50 (95% confidence interval 2.38–5.16) is taken from an earlier publication.^53^ As discussed elsewhere,^54^ this odds ratio could be a serious overestimate, due to inadequate control of confounding factors. This publication also shows that, in both the United States and United Kingdom, smoking prevalence in 2014–2016 has fallen faster than predicted by the preceding trend. Had a substantial gateway effect existed, one would expect the opposite.

## Discussion

Estimating the population health impact of MRTP introduction is challenging. Even limiting attention to cigarettes and one MRTP, many potential adverse and beneficial effects might occur. This is illustrated in Table 4, which lists the various changes in product use that may happen if an MRTP is introduced, and the resultant change in effective dose (scored as 0 for no use, 1 for smoking, *F* for exclusive MRTP use, and *G* for dual use). The effective dose is proportional to the increase in risk associated with product use. The main possible adverse effects are that some individuals who would otherwise not smoke do so following MRTP uptake (outcome A4 in Table 4), and that some smokers who would otherwise quit may switch to MRTP instead (outcome D2). The effects on the population of the different adverse and beneficial effects depend not only on their relative frequency, but also on the relative magnitude of their effects. Assuming *F* is small, the beneficial effect of outcomes involving a change from 1 to *F* are greater than the adverse effect of outcomes involving a change 0 to *F*. Table 4 shows that general inferences about effects of MRTP introduction cannot be made. Even assuming known *F*- and *G*-factors, introducing MRTP in a population with no smokers will have an adverse effect, while introducing it into a population with 100% smokers will be beneficial (unless dual use involves greater risk than cigarette-only smoking). Estimation needs conducting in plausible situations. However, this involves considerable uncertainties. These include lack of precise knowledge of the uptake rate of the MRTP (and all associated TTPs), the *F*- and *G*-factors, and full details of how complex patterns of smoking and MRTP use relate to risk. Not knowing, in the null scenario, the proportions of never tobacco users destined (without the MRTP) to initiate smoking, of smokers destined to quit, or of quitters destined to re-initiate, is also problematic.

**Table 4.** Theoretical Adverse and Beneficial Effects of Introducing an MRTP With *F*-Factors of *F* for MRTP Use and *G* for Dual Use

Initial status	Action if no MRTP		Actual action	Effective dose if MRTP not introduced	Effective dose if MRTP introduced	Effect^a^
Never tobacco	Does not initiate	A1.	Does not initiate tobacco	0	0	Nil
		A2.	Becomes MRTP-only user	0	F	Adverse
		A3.	Becomes dual user	0	G	Adverse
		A4.	Becomes cig-only smoker (via prior MRTP uptake)	0	1	Adverse
Never tobacco	Initiates	B1.	Becomes cig-only smoker	1	1	Nil
		B2.	Becomes MRTP-only user	1	F	Benefit
		B3.	Becomes dual user	1	G	Benefit?
Cig smoker	Does not quit	C1.	Stays cig-only smoker	1	1	Nil
		C2.	Becomes MRTP-only user	1	F	Benefit
		C3.	Becomes dual user	1	G	Benefit?
		C4.	Becomes quitter (via prior MRTP uptake)	1	0	Benefit
Cig smoker	Quits	D1.	Quits	0	0	Nil
		D2.	Becomes MRTP-only user	0	F	Adverse
		D3.	Becomes cig-only smoker (via prior MRTP uptake)	0	1	Adverse
Quitter	Does not re-initiate	E1.	Stays a quitter	0	0	Nil
		E2.	Becomes MRTP-only user	0	F	Adverse
		E3.	Becomes dual user	0	G	Adverse
		E4.	Becomes cig-only smoker (via prior MTRP uptake)	0	1	Adverse
Quitter	Re-initiates	F1.	Becomes cig-only smoker	1	1	Nil
		F2.	Becomes MRTP-only user	1	F	Benefit
		F3.	Becomes dual user	1	G	Benefit?

MRTP = modified risk tobacco product.

^a^The effective dose is taken to be proportional to the increase in risk associated with use. Thus, assuming that exclusive current cigarette smoking increases the effective dose by 1 unit, the *F*-factor (taken to be <1) is the unit increase in the effective dose for exclusive current MRTP use and the *G*-factor is the unit increase in the effective dose for current dual use.

Models can only approximate reality, and as they consider either future projections or what might theoretically have happened with past introduction of an MRTP, conventional goodness-of-fit testing of the results is not possible.

In the models described here:

• Only two products are generally considered
• Only overall mortality, or mortality from specific diseases is considered; morbidity not being directly investigated
• Demographic variables (race, socioeconomic status, etc.) are not included as risk factors
• The ability to model complex exposure histories is limited
• Only direct effects are considered: reductions in passive smoking, and the possibility that switching to the MRTP may affect other disease risk factors such as alcohol consumption, are ignored
• Unverifiable assumptions are made about the future
• There is limited available current input data

These limitations are difficult or impossible to avoid. Thus, allowing, in the alternative scenario, TTPs involving use of multiple products subdivided by extent of use could produce huge numbers of unverifiable “guesstimates.” Similarly, estimating the effect of future changes in factors other than tobacco involves many doubtful assumptions. However, some limitations may better be considered as features. Thus, whether overall mortality or mortality by cause is considered depends on the model’s purpose. Provided the diseases considered cover most smoking-attributable deaths, it may be useful to differentiate effects by cause.

Numerous aspects of specific models might be considered limitations in those they apply to, as summarized in Table 5, where all models have three or more of the limitations listed. One problem is that good epidemiological evidence is lacking on the effects of introducing MTRPs. E-cigarettes have been marketed for about 10 years, and no reliable results are yet available on risk in users of heart disease and stroke, major diseases where risk declines rapidly on quitting^47,49^ and could well have changed rapidly on switching to some MRTPs. A recent publication^55^ claimed e-cigarette use increases risk of a myocardial infarction, but based on a cross-sectional study where the sequence of events was unrecorded, so many cases could have occurred before e-cigarette initiation. Many models assume the *F*-factor is very low, 0.1, or less. Better evidence is needed, from prospective studies or well-designed case–control studies, to more reliably estimate it, and hence estimate more precisely long-term effects of MRTP introduction on mortality.

**Table 5.** Possible Limitations Present by Approach

Limitation	Study									
	ALCS1	ALCS2	BAT	JTI	PMI	RJR	FDA	LEVY1	LEVY2	UM
Deaths not removed during follow-up					✓ ^a^			✓	✓	
Current smokers not subdivided by amount smoked	✓	✓	✓		✓	✓	✓	✓	✓	✓
Former smokers not subdivided by time quit									✓	
Re-initiation not allowed for in null scenario		✓		✓				✓	✓	
Re-initiation not allowed for in alternative scenario				(✓)^b^				✓	✓	
Do not fully allow for initiation of both products				✓			✓	✓	✓	
Former smoker TTPs independent of previous product used				✓	✓			✓		✓
TTPs not age-dependent								✓		N/A
Decreased cigarette consumption or risk in dual users is not accounted for	✓	✓	✓			✓	✓		N/A	
Disease risks do not depend on the full tobacco history			✓				✓	✓	✓	
Age-specific risk in never tobacco users is assumed invariant over the follow-up period	✓	✓	✓	✓		✓	✓	✓	✓	✓
Results are not presented by age			✓	✓						✓
Results are not presented by gender	✓		✓						✓	✓

N/A = not applicable; TTP = tobacco transition probability.

^a^But a correction for differential survival is described.

^b^Allows only for re-initiation as MRTP.

Clearly, assumptions used in modeling should be plausible, and where possible based on prior information. However, uncertainty estimates should be presented, with sensitivity analyses illustrating how conclusions depend on key parameters. Given adequate allowance for transition rates between tobacco groups, which preferably should depend on age, gender, smoking history and period of MRTP introduction, and given the model to determine risk depends adequately on smoking history, one can gather useful insight into how the differing assumptions affect changes in mortality following MRTP introduction. The analysis and comparisons presented here elucidate the similarities and differences in structure, assumptions, and input data used by modelers in disparate settings.

Only the NIH model, focusing on e-cigarettes, suggests likely harm from MRTP introduction. As discussed, this model pessimistically assumes a large gateway effect, and that e-cigarette use reduces the likelihood of quitting among smokers interested in so doing.

It is reassuring that nearly all the approaches—based on differing methods and affected by differing strengths and limitations—suggest that MRTP introduction would have a beneficial population health impact. Extra insight may be gained by comparing different modeling approaches using the same input data (country, time period, tobacco groups, TTPs, smoking relative risks, and *F*- and *G-*factors), but this will probably only underline the general conclusion.

## Conclusions

Despite methodological differences, most modelers conclude MRTP introduction likely has a beneficial impact. However, the predictions are dependent on various factors, including the rate of MRTP uptake and the assumed values of the *F*- and *G*-factors, for which epidemiological evidence is currently lacking. Further model development, supplemented by results from well-designed epidemiological studies, should enable more accurate prediction of effects of MRTP introduction.

## Supplementary Material

A Contributorship Form detailing each author’s specific involvement with this content, as well as any supplementary data, are available online at https://academic.oup.com/ntr.

Supplementary data are available at *Nicotine & Tobacco Research* online.

ntaa102_suppl_Supplementary_File_1

ntaa102_suppl_Supplementary_File_2

ntaa102_suppl_Supplementary_Taxonomy_Form
