# Supplementary material for: Estimating the Population Health Impact of Recently Introduced Modified Risk Tobacco Products: A Comparison of Different Approaches
Source: Nicotine Tob Res. 2020 Jun 4;23(3):426–37. doi: 10.1093/ntr/ntaa102 (PMC7885777; doi:10.1093/ntr/ntaa102)
Supplement: ntaa102_suppl_Supplementary_File_1 [file ntaa102_suppl_supplementary_file_1.docx]

“Estimating the population health impact of recently introduced modified risk tobacco products: a comparison of different approaches”

By Peter N Lee et al.

Supplementary File 1 (Tables)

Date: March 2020

This file consists of five tables.

Supplementary Tables 1 and 2

These describe the same list of 48 characteristics of the ten cohort-based models (See Table 1 of the paper for the abbreviations used). Supplementary Table 1 gives information relating to the six models sponsored by the tobacco industry (ALCS1, ALCS2, BAT, JTI, PMI and RJR), while supplementary file 2 considers the four models sponsored by public funds (FDA, LEVY1, LEVY2 and UM). The information given for each model is the same. The first set of characteristics (from “References” to “Quantitative conclusions from main model”) are followed by information on limitations of the models (those with “No” entered have limitations) and information on sources of the input data used. Note that references to the sources are not given in this note, being available from the source papers. Other abbreviations used are as follows:

CC = conventional cigarettes, CDC = Centers for Disease Control and Prevention, CISNET = Cancer Intervention and Surveillance Modelling Network, CPD = cigarettes per day, COPD = chronic obstructive pulmonary disease, CPS = Cancer Prevention Study, IHD = Ischaemic heart disease, LMF = Linked Mortality Files, MRTP = modified risk tobacco product, NGP = new generation product, NHIS = National Health Interview Survey, NLMS = National Longitudinal Mortality Survey, NSDUH = National Survey on Drug Use and Health, ONS = Office for National Statistics, PATH = Population Assessment of Tobacco and Health, RRP = reduced risk product, SAMHSA = Substance Abuse and Mental Health Services Administration, ST = smokeless tobacco, TTP = tobacco transition probabilities, USDHHS = US Department of Health and Human Services and VNP = vaporized nicotine product.

Supplementary Tables 3 and 4

These tables summarize the tobacco transition probabilities used in the 10 cohort-based models in the Null Scenario (Table 3) and in the Alternative Scenario (Table 4). They show which of the possible transitions are considered in the different models and give details of factors affecting the transitions.

Supplementary Table 5

This table gives fuller detail for each model on how the population health impact was estimated from the tobacco histories developed via the tobacco transition probabilities.

#### Supplementary Table 1 : Characteristics of the six cohort-based tobacco industry sponsored models

|  | **Model** |  |  |  |  |  |
| --- | --- | --- | --- | --- | --- | --- |
| **Characteristic** | **ALCS1** | **ALCS2** | **BAT** | **JTI** | **PMI** | **RJR** |
| References | Black et al. (2018); Muhammad-Kah et al. (2019) | Black et al. (2018) | Hill and Camacho (2017) | Poland and Larroque (2018); Poland and Teischinger (2017) | Djurdjevic et al. (2018a); Djurdjevic et al. (2018b); Lee et al. (2017); Weitkunat et al. (2015) | Bachand and Sulsky (2013); Bachand et al. (2018) |
| Sponsors | Altria Client Services | Altria Client Services | British-American Tobacco | Japan Tobacco International | Philip Morris International | R J Reynolds |
| Summary of method | “Our model consists of a Markov compartmental model based on cohorts starting at a defined age and followed up to a specific age accounting for 29 tobacco-use states based on a cohort members transition pathway. The Markov model is coupled with statistical mortality models and excess relative risk ratio estimates to determine survival probabilities from use of smokeless tobacco. Our model estimates the difference in premature deaths prevented by comparing Base Case (“world-as-is”) and Modified Case (the most likely outcome given that a modified risk claim is authorized) scenarios.” Muhammad-Kah et al. (2019) | Each agent has an associated set of attributes. In the ALCS agent-based model, attributes are updated at each time point and tracked throughout the simulation. Agent attributes include whether the agent is alive, age, gender, tobacco status at each year, including years of use and years since quit for each product. The model consists of two interlinked sub-models – transition and mortality (Adapted from  (Black et al., 2018)) | “A model based on system dynamics methodology was developed to project the potential effects of a new nicotine product at a population level. A model representing traditional smoking populations (never, current and former smokers) and calibrated using historical data was extended to a two-product model by including electronic cigarettes use statuses. Smoking mechanisms, such as product initiation, switching, transition to dual use, and cessation, were represented as flows between smoking statuses (stocks) and the potential effect of smoking renormalisation through a feedback system. Mortality over a 50-year period (2000-2050) was the health outcome of interest, and was compared between two scenarios, with and without e-cigarettes being introduced.” Hill and Camacho (2017) | A Monte Carlo simulation model of individual tobacco product use histories and deaths which accounts for graded excess risk changes. Data are tracked by individual in nodes subdivided by age, gender, cigarettes per day and tobacco use history. It was run repeatedly over a wide range of inputs for a hypothetical MRTP with each run simulating 1 million people. (Adapted from  (Poland and Larroque, 2018)) | “Simulated samples start in 1990 with a US representative smoking prevalence. Individual tobacco histories are updated annually until 2010 using estimated probabilities of switching between never/current/former smoking where the RRP is not introduced, with current users subdivided into cigarette/RRP/dual users where it is. RRP-related mortality reductions from lung cancer, IHD, stroke and COPD are derived from the histories and the assumed relative risks of the RRP.” Lee et al. (2017) | “The model sorts the population into age and exposure categories, and applies the appropriate mortality rates to each category. The model tracks individual exposure histories, and estimates, at the end of each modeled age category, the number of survivors in the two exposure scenarios (base case and counterfactual), and the difference between them. Markov Chain Monte Carlo techniques are used to estimate the variability of the results.” Bachand and Sulsky (2013) |
| Product considered | MRTP- Smokeless Tobacco Product (following introduction of a modified risk claim) | MRTP | New nicotine product (or new generation product) | MRTP | MRTP | MRTP |
| Countries applied to | US | US | UK | US | US | US |
| Health endpoints | All-cause mortality  Years of life lost | All-cause mortality  Years of life lost | All-cause mortality | All-cause mortality | Mortality from lung cancer, COPD, IHD and stroke. Years of life lost | All-cause mortality  Years of life lost  Quality of life |
| Results by age | Yes | Yes | No | No | Yes | Yes |
| Results by gender | Yes | Yes | No, but uses gender-specific inputs | No, but uses gender-specific inputs | Yes | Yes |
| Name of null scenario | Base case | Base case | Core | Cigarette | Null | Base case |
| Name of alternative scenario | Modified case | Modified case | NGP | MRTP | Alternative | Counterfactual |
| Age at start | 13 years (initially never smokers) | All | All | 18+ years | 10-79 years | 12 years (initially never smokers) |
| Population added to over time | No | Births and immigrations added | Births and immigrations added | New 18 year olds added | No | No |
| Population subtracted from over time | Deaths are removed | Deaths and emigrations are removed | Deaths and emigrations are removed | Deaths are removed | No, but a correction or differential survival is described | Deaths are removed |
| Use random numbers to determine transitions between product status for individuals | No; only groups are transitioned, but uncertainty is taken into account | Yes | No; only groups are transitioned | Yes | Yes | No; only groups are transitioned, but uncertainty is taken into account |
| Follow-up period | 60 years (2015-2075) | 60 years (2000-2060) | 50 years (2000-2050) | 88 years (2012-2100) | 20 years (1990-2010) | From age 13, until all die |
| Smoking habits in Null Scenario | Never tobacco, current cigarette smoker, former cigarette smoker | Never tobacco, current cigarette smoker, former cigarette smoker | Never cigarettes, current cigarette smokers, former cigarette smokers (by time quit) | Never smoked, current cigarette smokers, former cigarette smokers (by time quit) | Never conventional cigarettes (CC), current CC, former CC (by time quit) | Never base case product, current base case product, former base case product |
| TTPs considered in Null Scenario | Initiation, cessation, re-initiation. The rates may vary by age, time quit, and duration of smoking | Initiation, cessation. The rates may vary by age, gender, time quit, and current and past use of tobacco | Initiation, cessation, re-initiation. The rates may vary by age, gender, and time quit | Initiation (only at age 18), cessation. The rates may vary by age and gender | Initiation, cessation, re-initiation. The rates may vary by age, gender, time quit, previous quitting and length of follow-up | Initiation, cessation, re-initiation (only once). The rates may vary by age, gender, current age and past use of tobacco |
| Tobacco habits in Alternative Scenario | Never tobacco, current cigarettes only, current dual use, current MST only, former cigarettes only, former dual use, former MST only | All nine groups from combination of never/current/former cigarettes by never/ current/former MRTP | Never tobacco, current cigarettes only, current dual use, current RRP only, former cigarettes only, former dual user, former RRP only | Never either product, current cigarettes only, current MRTP only, current dual use, former use of either or both | Never tobacco, current CC only, current RRP only, current dual use only, former use of either or both | All nine groups from combination of never/current/former cigarettes by never/ current/former MRTP |
| TTPs considered in Alternative Scenario | Initiation to either product (not to dual use), quitting from either product (not from dual use), re-initiation from all three former groups to either product (not to dual use), all switches between current groups | Initiation to either product (not to dual use), quitting from either product (not from dual use), re-initiation from former smoking to the other product, quitting from all current groups except dual use ,all switches between current groups | Initiation to all three current groups, quitting from all three current groups, re-initiation from all three former groups to any current group, all switches between current groups | Initiation to either product (not to dual use), quitting from either product (not from dual use), re-initiation (only as MRTP), all switches between current groups | Initiation to all three current groups, quitting from all three current groups, re-initiation to any current group, all switches between current groups | Initiation to either product (not to dual use), quitting from all three current groups, re-initiation to either product (not to dual use), switches between products or to dual use (not from dual use) |
| Validation of TTPs | No validation of projected prevalence estimates. | Model projected smoking prevalences by gender and age from 2000-2015 were compared with estimates from CDC and NHIS. | For Null Scenario, projections for 2010-2050 were compared with forecasts from ONS. | The model was calibrated with US Census Bureau population data and projections, as well as NSDUH data on smoking prevalence and cigarettes per day from 2002-2009 reserving 2010-2012 data for validation. | For the Null Scenario projections of smoking distributions from 1990 to 2010 were compared with actual data. | Projected survival over time compared to population life tables. |
| F-factors | 0.09 | 0.05 | 0.05 | 0.04 to 0.10 | 0.20 | 0.08 |
| G-factors | 1 | 1 | 1 | 1 + F but account was made for a 42% reduction in cigarette consumption in current smokers | (1 + F)/2 in many analyses; also 0.4 to 2.0 | 1 |
| Allows for environmental tobacco smoke | No | No | No | No | No | No |
| Allows for risk factors other than age, gender and smoking | No | No | Yes – effect of smoking normality on initiation and quitting rates | No | No | No |
| Selected quantitative conclusions from presented examples | “Our model predicts an estimated 93,000 premature deaths would be avoided over a 60-year period upon authorization of a modified risk claim. Our sensitivity analyses using various reasonable ranges of input parameters do not indicate any scenario under which the net benefit could be offset entirely.” Muhammad-Kah et al. (2019) | “Our model suggests overall net benefit of introducing E-cigarettes into the US market, as indicated by a reduction in smoking prevalence and a decrease in all-cause mortality (~629,000 premature deaths prevented) in the US population compared to the status quo. The sensitivity analysis indicates that relatively large changes to the ERR of an E-cigarette would still predict a net benefit to the population under our defined scenario.” Black et al. (2018) | “The results suggest that by 2050, smoking prevalence in adults was 12.4% in the core model and 9.7% (including dual users) in the counterfactual. Smoking-related mortality was 8.4% and 8.1%, respectively. The results suggested an overall beneficial effect from launching e-cigarettes and that system dynamics could be a useful approach to assess the potential population health effects of nicotine products when epidemiological data are not available.” Hill and Camacho (2017) | “Hypothetical MRTP would nearly halve smoking prevalence and avoid 1.1M premature deaths by 2050 (5.6M by 2100) in base case.” Poland and Larroque (2018) | “The mortality reduction is proportional to the dose reduction, increasing rapidly with time of follow-up. Plausible increases in re-initiation or dual users’ consumption, or decreased quitting by smokers would not eliminate the drop.” Lee et al. (2017) | “In the counterfactual scenario, where 2% of current smokers who would have continued to smoke instead switched completely to and then continued to use an MRTP, there was a statistically significant survival benefit of 3,127 additional survivors (95% PI: 2,751–3,508) compared to the base case at the end of age category 68–72, a 0.10-year increase in LE at age 18 years, and a 0.07-year increase in QALE at age 18 years.” Bachand et al. (2018) |
| LIMITATIONS (Those with limitations are entered as No) |  |  |  |  |  |  |
| Removals during follow-up | Yes, deaths only | Yes; deaths and emigrations | Yes; deaths and emigrations | Yes; deaths only | No | Yes, deaths only |
| Current smokers subdivided by amount smoked | No | No | No | Yes | No | No |
| Former smokers subdivided by time quit | Yes | Yes | Yes | Yes | Yes | Yes |
| Re-initiation allowed for in null scenario | Yes | No | Yes | No | Yes | Yes |
| Re-initiation allowed for in alternative scenario | Yes | Yes | Yes | Yes (only as MRTP) | Yes | Yes |
| Fully allow for initiation of both products in the same time period | No | No | Yes | No | Yes | Yes |
| Former smoker TTPs dependent on previous product used | Yes | Yes | Yes | No | No | Yes |
| TTPs age-dependent | Yes | Yes | Yes | No | Yes | Yes |
| Decreased cigarette consumption or risk in dual users is not accounted for | No | No | No | Yes | Yes | No |
| Mortality/ Disease risk depends on the full tobacco history | Yes | Yes | No | Yes | Yes | Yes |
| Age-specific risk in never tobacco users varies over the follow-up period | No | Yes | No | No | Yes | No |
| Results presented by age | Yes | Yes | No | No | Yes | Yes |
| Results presented by gender | Yes | Yes | No, but use gender-specific inputs | No, but use gender-specific inputs | Yes | Yes |
| SOURCES |  |  |  |  |  |  |
| Sources of initial population | Population starts as never smokers aged 13. | U.S. Census National Population Estimates for 2000. U.S. cigarette smoking status (current smoker, former smoker, and never tobacco user) by gender and age from 2000 NHIS data | Initial population from Mid-2001 Population Estimates (ONS). Initial smoking prevalence from General Lifestyle Survey (ONS). Initial former smoker quit time distribution from Smoking-related Behaviour and Attitudes (2009/09) (ONS) and Smoking Cessation in England 2010 (West) | 2012 population data from US Census Bureau. Smoking status based on 2012 NSDUH survey. Current and former smokers were assigned an average CPD level and former smokers a number of years since quitting, also based on this survey. | Baseline distribution of age and gender from UN. Distribution by smoking status based on International Smoking Statistics. Distribution of quitters by age at quitting based on NHIS data for 2006. | Population starts as never smokers aged 12. |
| Sources of immigration and emigration rates | N/A | U.S. Census National Population Projections 2008 | Sources not described | N/A | N/A | N/A |
| Sources of birth rates | N/A | U.S. Census National Population Projections 2008 | National Population Projections 2012 | Population growth was specified by the annual sizes of the new 18 year-old subpopulation as projected by the US Census Bureau | N/A | N/A |
| Sources of death rates | The Kaiser-Permanente Cohort Study provided mortality data for never, current and former tobacco users by age, gender, duration of smoking and duration of quitting. KP data was adjusted to reflect the 2000 U.S. population (National Vital Statistics). | The Kaiser-Permanente Cohort Study provided mortality data for never, current and former tobacco users by age, gender, duration of smoking and duration of quitting. KP Study mortality data was aligned with the 2000 Human Mortality Database (HMD). | National Life Tables, UK 1980-82 to 2012-14 | All-cause mortality rates were modeled by age group, gender, and smoking status. Background all-cause mortality for never-smokers, and relative risks (RRs) of death for smokers, by age group and gender were taken from Thun et al. who reported on “contemporary cohorts” of 956,756 subjects with mean age 66 years but excluding ages <55 (Table S1). USDHHS provided RR for 35-54 year old men and women (Table S2), and never-smoker death rates for 35-54 year old men and women, not supplied in either reference, were taken from Woloshin et al. Death rates were then adjusted to approximate those of the US Census Bureau’s 2012-2060 population projections. Poland and Teischinger (2017) | N/A | The Kaiser-Permanente Cohort Study, adjusted for the 2000 US census, provided mortality data for never, current and former tobacco users by age, gender, duration of smoking and duration of quitting. |
| Sources of TTPs for null scenario | Nationally representative TTPs were estimated from review of six US studies on transitions between smokeless tobacco and cigarette use. Tam et al. (2015) | Initiation and cessation rates were taken to be those of year 2000 derived from NHIS and CISNET. | Initiation, cessation and re-initiation rates were derived from Multiple UK sources as shown in Table 1 of the source paper. | The initiation rate was taken as the cigarette smoking prevalence percentage at age 18, plus any increases at age 19-20, based on NSDUH data. It was modeled to decline linearly based on 2002-2009 data, but rates were fixed from 2012 onwards. Cessation rates were based on NHIS data and modeled as exponential increases in the cessation rate for the most recent birth cohort provided, 1970. The model assumed smoking cessation is permanent, “to avoid complex modeling of relapses”. As the rates based on the NHIS data may be too high for permanent cessation, they were “tuned down” to fit smoking prevalence data by gender and age group. | Initiation, cessation and re-initiation rates were derived “from educated guesses which produced not unreasonable estimates of current and former smoking during follow-up”. Comparison with rates from a representative US population led to the re-initiation rates being increased. | For the illustrative examples, initiation rates were based on 2009 rates from NSDUH, and cessation rates were based on 2005-2008 rates from SAMHSA. Uncertainty in the rates was modeled using truncated normal variables with means equal to the respective estimates and standard deviations equal to 0.01. |
| Sources of TTPs for alternative scenario | From a “Claim comprehension and intentions study” in which 5,871 participants were asked about their behavioural intentions and intent to purchase. | Estimated from changes in Waves 1 and 2 of the PATH study Cessation rates were taken to be those of year 2000 derived from NHIS and CISNET. | Based on assumptions | Based on assumptions | The TTPs were developed to produce a defined distribution of current use 10 years after MRTP introduction; and to reflect the fact that younger people may be less likely to switch, because of cost. The sum of these initiation TTPs was set to be equal to that in the Null Scenario, and similarly for re-initiation. Each of the quitting TTPs was set equal to that in the Null Scenario. | For the illustrative examples, based on assumptions |
| Sources of F-factors | The excess risk of current ST users relative to current smokers, and of former ST users relative to former cigarette smokers was estimated from the National Health Interview Survey (NHIS) and the National Longitudinal Mortality Survey (NLMS), linked to mortality information from the National Death Index (NDI) of the National Center for Health Statistics (NCHS). Fisher et al. (2019) | F was taken as 0.05 as estimated by an international expert panel convened by the Independent Scientific Committee on Drugs (ISCD) ( (Nutt et al., 2014)), also reported by Public Health England. | E-Cigarettes were taken to be 5% as harmful as traditional cigarettes based on the report from Public Health England referred to under ALCS2. | No specific sources were used. | Estimates were derived from aerosol, chemistry, toxicology, pharmacokinetic, biomarker and clinical data, but a wide range of F values was tested due to considerable uncertainty. | For the illustrative examples, a value of 0.08 was based on a consensus estimate for the mortality risk of a low-nitrosamine ST product relative to CCs. Uncertainty in this value was accounted for by modeling F as a left-truncated normal random variable with mean 0.08 and SD 0.01. |
| Sources of G-factors | Dual users were assumed to have the same risk as exclusive cigarette smokers based on published literature. | Dual use was assumed to give the same risk as exclusive cigarette smoking. | G was assumed to be the maximum of 1 and F | G was assumed to be the sum of 1, times a non-linear adjustment for reduced cigarette smoking based on the literature, and F. | A wide range of values was tested. The main estimate of (1+F)/2 assumes dual users smoke 50% cigarettes and 50% RRP. | Dual use was assumed to give the same risk as exclusive cigarette smoking. |
| Sources of current smoking RRs | These were derived from Kaiser- Permanente Cohort Study, adjusted to take into account the subjects all having health insurance and 2000 U.S. population (National Vital Statistics) | These were derived from Kaiser- Permanente Cohort Study, adjusted to take into account the subjects all having health insurance and 2000 Human Mortality Database (HMD) | These were taken on a report on total mortality and smoking in UK from P.N. Lee Statistics and Computing Ltd. | See sources of death rates. Also RRs by CPD were taken from studies by Thun et al. | These were taken from published meta-analyses for lung cancer and COPD, and from provided meta-analyses for heart disease and stroke. | These were derived from the Kaiser-Permanente Cohort Study and the 2000 US Census. |
| Sources of quitting half lives | Former smoker RRs were derived as for current smoking RRs. | Former smoker RRs were derived as for current smoking RRs. | Former smoker RRs were based on the same report as for current smoking. The half-lives used were as reported by Weitkunat et al. (2015) for the PMI model. | See sources of death rates for sources of former smoker RRs. | The half-lives were taken from published meta-analyses for all four diseases. | Former smoker RRs were derived as for current smoking RRs. |

#### Supplementary Table 2 : Characteristics of the four cohort-based models sponsored by public funding

|  | **Model** |  |  |  |
| --- | --- | --- | --- | --- |
| **Characteristic** | **FDA** | **LEVY1** | **LEVY2** | **UM** |
| References | Apelberg et al. (2018); Vugrin et al. (2015) | Levy et al. (2017) | Levy et al. (2018) | Warner and Mendez (2019) |
| Sponsors | US Food and Drug Administration | National Institute on Drug Abuse, Cancer Intervention and Surveillance Modeling Network, National Cancer Institute | National Institute on Drug Abuse, Cancer Intervention and Surveillance Modeling Network, National Cancer Institute | University of Michigan |
| Summary of method | “We present a multi-state, dynamical systems population structure model that can be used to assess the effects of tobacco product use behaviors on population health. The model incorporates transition behaviors, such as initiation, cessation, switching, and dual use, related to the use of multiple products. The model tracks product use prevalence and mortality attributable to tobacco use for the overall population and by gender and age group. The model can also be used to estimate differences in these outcomes between scenarios by varying input parameter values. We demonstrate model capabilities by projecting future cigarette smoking prevalence and smoking-attributable mortality and then simulating the effects of introduction of a hypothetical new lower-risk tobacco product under a variety of assumptions about product use.” Vugrin et al. (2015)  Apelberg et al. (2018) uses the Vugrin model to consider the effects of lowering the nicotine content of cigarettes to minimally addictive levels, the product then legally available not having any reduced risk in cigarette smokers who switch to it, but causing beneficial health effects indirectly, since some cigarette smokers would quit or switch to a lower risk product. | “The public health impact of VNP use is modeled in terms of how it alters smoking patterns among those who would have otherwise smoked cigarettes and among those who would not have otherwise smoked cigarettes in the absence of VNPs. The model incorporates transitions from trial to established VNP use, transitions to exclusive VNP and dual use, and the effects of cessation at later ages. Public health impact on deaths and life years lost is estimated for a recent birth cohort incorporating evidence-informed parameter estimates.” Levy et al. (2017) | “A Status Quo Scenario, developed to project smoking rates and health outcomes in the absence of vaping, is compared with Substitution models, whereby cigarette use is largely replaced by vaping over a 10-year period. We test an Optimistic and a Pessimistic Scenario, differing in terms of the relative harms of e-cigarettes compared with cigarettes and the impact on overall initiation, cessation and switching. Projected mortality outcomes by age and gender under the Status Quo and E-Cigarette Substitution Scenarios are compared from 2016 to 2100 to determine public health impacts.” Levy et al. (2018) | “Using a dynamic model that tracks the US adult population’s smoking status and smoking-related deaths over time, we simulate the effects of vaping-induced smoking initiation and cessation on life-years saved or lost to the year 2070. The base case assumes that vaping annually increases smoking initiation by 2% and smoking cessation by 10%. Sensitivity analyses raise the initiation rate increase to 6% while decreasing the cessation rate increase to 5%. Sensitivity analyses also test vaping’s reducing the health benefits of quitting smoking by 10%.” Warner and Mendez (2019) |
| Product considered | New tobacco product | Vaporized nicotine product | E-cigarettes | E-cigarettes |
| Countries applied to | US | US | US | US |
| Health endpoints | All-cause mortality | All-cause mortality Years of life lost | All-cause mortality | Al- cause mortality Years of life lost |
| Results by age | Yes | Yes | Yes | No |
| Results by gender | Yes | Yes | No, but uses gender-specific inputs | No |
| Name of null scenario | Status quo | No-VNP | Status quo | Status quo |
| Name of alternative scenario | Hypothetical | VNP | Substitution | Base case |
| Age at start | All | 15 years (initially never smokers) | 15-99 years | 18+ years |
| Population added to over time | Births and immigrations added | No | No | New 18 year olds added |
| Population subtracted from over time | Deaths and emigrations removed | No | No | Deaths removed |
| Use random numbers to determine transitions between product status for individuals | No; only groups are transitioned | No; only groups are transitioned | No; only groups are transitioned | No; only groups are transitioned |
| Follow-up period | 84 years (2016-2100) and 50 years (2000-2050) | 71 years (2012-2083) | 84 years (2016-2100) | 60 years (2010-2070) |
| Smoking habits in Null Scenario | For each product considered, the population is divided into never, current and former users. Thus, with two products there are nine groups. | The initial never smoker population is subdivided into those who would have become a cigarette smoker in the absence of VNPs (A) and those who would not (B). In those not trying VNPs, group A become current cigarette smokers, and group B does not. | Never cigarettes, current cigarettes, former cigarettes | Never cigarettes, current cigarettes, former cigarettes |
| TTPs considered in Null Scenario | Initiation, cessation, re-initiation. The rates may vary by age, gender, current and past use of tobacco. | Initiation (only to age 25), cessation, re-initiation (only to age 25). The rates may vary by gender. | Initiation, cessation. The rates may vary by gender. | Initiation (only at age 18), cessation. The rates are independent of age and gender. |
| Tobacco habits in Alternative Scenario | All nine groups from combinations of never/current/ former cigarettes by never/current/ former MRTP. | Never tobacco, current CC only, current RRP only, current dual use only, former use of either or both. | Never tobacco, current cigarettes only, current e-cigarettes only, former cigarettes only and former e-cigarettes only. | Never, current and former cigarettes. (E-cigarettes are only relevant to the effect they have on cigarette initiation and cessation.) |
| TTPs considered in Alternative Scenario | Initiation to current single and dual product use (with no former use); quitting from all five current groups; two re-initiations and six switches. (See note in Table 3 of the paper to which this is a Supplementary File.) | LEVY1 subdivides never smokers initially by whether they would (A) or would not (B) have started smoking in the absence of e-cigarettes, with A and B further subdivided by whether they try (1) or not try (2) VNPs. A1 and B1 are then subdivided by whether they try, then quit e-cigarettes or continue to use them. Each subgroup ends up as long-term users of never tobacco, single product, or dual use. With long-term use, no further transitions occur. | LEVY2, concerned with replacing cigarettes with e-cigarettes, allows initiation only with e-cigarettes in the alternative model, quitting rates being as in the null scenario. Re-initiation and switching are irrelevant. | Initiation only as smoking; cessation only from smoking; no re-initiation or switching. |
| Validation of TTPs | Model projections of smoking prevalence by gender and age for 2000-2012 were compared with estimates from CDC and NHIS. | None stated but the comparison described in LEVY2 would presumably apply. | Initiation and cessation rates projected from 1965-2012 data were validated by comparing smoking prevalence projections to 2010 against observed rates. | None |
| F-factors | 0.25 (range 0.01-0.50) in Vugrin paper. N/A in Apelberg paper. | 0.05 | 0.05 | 0 (but smokers quitting following e-cigarette use lose 10% of the mortality reduction from quitting). |
| G-factors | 1 | 0.70 (and alternatives from 0.50 to 1.00) | N/A | 1 |
| Allows for ETS | No | No | No | No |
| Allows for risk factors other than age, gender and smoking | No | No | No | No |
| Quantitative conclusion from main model | “We demonstrate that potential benefits from cigarette smokers switching to the lower-risk product can be offset over time through increased initiation of this product. Model results show that population health benefits are particularly sensitive to product risks and initiation, switching, and dual use behaviors.” Vugrin et al. (2015) | “Based on current use patterns and conservative assumptions, we project a reduction of 21% in smoking-attributable deaths and of 20% in life years lost as a result of VNP use by the 1997 US birth cohort compared to a scenario without VNPs. In sensitivity analysis, health gains from VNP use are especially sensitive to VNP risks and VNP use rates among those likely to smoke cigarettes.” Levy et al. (2017) | “Compared with the Status Quo, replacement of cigarette by e-cigarette use over a 10-year period yields 6.6 million fewer premature deaths with 86.7 million fewer life years lost in the Optimistic Scenario. Under the Pessimistic Scenario, 1.6 million premature deaths are averted with 20.8 million fewer life years lost. The largest gains are among younger cohorts, with a 0.5 gain in average life expectancy projected for the age 15 years cohort in 2016.” Levy et al. (2018) | “With base-case assumptions, the population gains almost 3.3 million life-years by 2070. If all people who quit smoking by vaping lose 10% of the benefit of quitting smoking, the net life-year gain falls to 2.4 million. Under worst-case assumptions, in which vaping increases smoking initiation by 6% and cessation by 5%, and vaping-induced quitters lose 10% of the health benefits, the population gains over 580 000 life-years.” Warner and Mendez (2019) |
| LIMITATIONS (Those with limitations are entered as No) |  |  |  |  |
| Removals during follow-up | Yes; deaths and emigrations | No | No | Yes; deaths only |
| Current smokers subdivided by amount smoked | No | No | No | No |
| Former smokers subdivided by time quit | Yes | Yes | Yes | Yes |
| Re-initiation allowed for in null scenario | No | No | No | Yes |
| Re-initiation allowed for in alternative scenario | Yes | No | No | Yes |
| Fully allow for initiation of both products | No | No | No | Yes |
| Former smoker TTPs dependent on previous product used | Yes | No | Yes | No |
| TTPs age-dependent | Yes | No | Yes | N/A |
| Decreased cigarette consumption or risk in dual users is not accounted for | No | Yes | N/A | Yes |
| Disease risks depend on the full tobacco history | No | No | No | Yes |
| Risk in never tobacco users varies over the follow-up period | Yes | No | No | No |
| Results presented by age | Yes | Yes | Yes | No |
| Results presented by gender | Yes | Yes | No, but uses gender-specific inputs | No |
| SOURCES |  |  |  |  |
| Sources of initial population | Initial population data from Census estimates. Initial smoking rates from NHIS. | Source of population data not stated. Initial smoking rates from NHIS. | 2016 population data from United Nations. Initial smoking rates from NHIS. | 2010 population from US Census data.  Initial smoking rates from NHIS. |
| Sources of immigration and emigration rates | US Census Bureau estimates | N/A | N/A | N/A |
| Sources of birth rates | US Census Bureau estimates | N/A | N/A | N/A |
| Sources of death rates | Never smoker rates were estimated from NHIS-Linked Mortality Files, with the RRs described below used to estimate smoker rates | Life tables for 1964-1980 cohorts from Holford et al were projected forward allowing for reduced death rates due to medical advances | Life tables for 1964-1980 cohorts from Holford et al were projected forward allowing for reduced death rates due to medical advances | Age-specific death rates from Statistical Abstracts of the US |
| Sources of TTPs for null scenario | Age-specific initiation and cessation rates were obtained from smoking histories reconstructed from NHIS data and assumed to be constant throughout the projection period | “To estimate smoking rates in the absence of VNP use, we analyze a cohort of current, former, and never smokers in the United States using data through 2012. The data were developed by applying an age-period-cohort statistical technique to National Health Interview Surveys (NHIS) from 1965–2012 while correcting for bias due to higher mortality among smokers. Since sustained VNP use was still low in 2012, the NHIS data are used to approximate cigarette smoking trends prior to VNPs.” Levy et al. (2017) | “Smoking rates are projected forward using age-specific and sex-specific initiation and cessation rates, and age-specific, sex-specific and smoking status-specific mortality rates. Holford et al developed the initiation and cessation rates by applying an age-period-cohort statistical model to data from the 1965–2012 National Health Interview Surveys while correcting for bias due to higher mortality among current and former smokers.” Levy et al. (2018) | Initiation rates were projected to 2028 from 2005-2014 NHIS data and then taken to be constant. Cessation rates were projected to 2028 from 1990-2014 data then taken to be constant. |
| Sources of TTPs for alternative scenario | Purely hypothetical in Vugrin paper. Based on expert projections in Apelberg paper. | The TTPs shown in Figure 1 of the source paper are described as “evidence based” with justification given in the text. | The Optimistic Scenario TTPs were based primarily on current use patterns in e-cigarettes; The Pessimistic Scenario TTPs involved vaping increases compared to Status Quo Scenario. | In the base-case model vaping was assumed to increase smoking initiation rates by 2% and cessation rates by 10% based on various sources. (See source publication.) |
| Sources of F-factors | Purely hypothetical | The 5% increase in risk was based on a multi- decision analysis. | The 5% increase in risk reflects published reports. | Vaping was treated as harmless, since UK data support individuals who quit smoking by vaping stopping vaping within a year or two. |
| Sources of G-factors | Purely hypothetical | “While some studies of VNP use report reductions in cigarette use of more than 50%, others indicate smaller reductions, especially among nondaily VNP users. A review found 75%–80% lower cigarette consumption among dual cigarette and snus users than among exclusive smokers.” Levy et al. (2017) | N/A | Vaping was treated as harmless, since UK data support individuals who quit smoking by vaping stopping vaping within a year or two. |
| Sources of current smoking RRs | Hazard ratios were estimated from NHIS-LMF data for 1997-2004 followed to 2006 | Death rates by smoking and gender came from CPS I and II | Death rates by smoking and gender came from CPS I and II | Death rates by smoking status came from CPS II |
| Sources of quitting half lives | Hazard ratios for former smokers (by age at cessation) were also estimated from NHIS-LMF data | Death rates for former smokers also came from CPS I and II | Death rates for former smokers also came from CPS I and II | Death rates for former smokers by time quit also came from CPS II |

#### Supplementary Table 3 : Tobacco transition probabilities in the null scenario

|  |  |  |  | Transition may vary by | | |
| --- | --- | --- | --- | --- | --- | --- |
| Model | Initiation^a^ | Cessation^b^ | Re-initiation^c^ | Age | Gender | Others |
|  |  |  |  |  |  |  |
| ALCS1 | Yes | Yes | Yes | Yes | No | Time quit, smoking duration |
| ALCS2 | Yes | Yes | No^d^ | Yes | Yes | Time quit, smoking duration, current and past use of tobacco |
| BAT | Yes | Yes | Yes | Yes | Yes | Time quit |
| JTI | Only at age 18 | Yes | No | Yes | Yes | No |
| PMI | Yes | Yes | Yes | Yes | Yes | Time quit, previous quitting, length of follow-up |
| RJR | Yes | Yes | Only once^d^ | Yes | Yes | Current and past use of tobacco |
| FDA | Yes | Yes | No^d^ | Yes | Yes | Current and past use of tobacco |
| LEVY1 | Only to age 25 | Yes | Only to age 25 | No | Yes | No |
| LEVY2 | Yes | Yes | No | Yes | Yes | No |
| UM | Only at age 18 | Yes | No | N/A | No | No |
|  |  |  |  |  |  |  |

Note: The six tobacco industry sponsored models are shown first. N/A = not applicable

^a^ From never smoker to current smoker

^b^ From current smoker to former smoker

^c^ From former smoker to current smoker

^d^ Analyses based on input data for “successful” cessation, so multiple re-initiation was unlikely

#### Supplementary Table 4 : Tobacco transition probabilities in the alternative scenario

| Model | Initiation^a^ | Cessation^b^ | Re-initiation^c^ | Switching^d^ | Comment |
| --- | --- | --- | --- | --- | --- |
|  |  |  |  |  |  |
| ALCS1 | Yes (2) – not directly to dual use | Yes (2) | Yes (4) – not to dual use | Yes (6) | - |
| ALCS2^g^ | Yes (2) – not directly to dual use | Yes (4) – not directly from dual use | Yes (1) – not directly to dual use | Yes (6) | Transition rates are based on established use and history of use |
| BAT | Yes (3) | Yes (3) | Yes (9) | Yes (6) | - |
| JTI | Yes (2) – not to dual use | Yes (2) – not from dual use | Yes (1) – only as MRTP | Yes (6) | - |
| PMI | Yes (3) | Yes (3) | Yes (3) | Yes (6) | - |
| RJR | Yes (2) – not to dual use | Yes (3) | Yes (2) – not to dual use. | Yes (4) – not from dual use to single product use | Many TTPs can only occur once, and none can occur more than twice^f^ |
| FDA^e^ | Yes (3) | Yes (5) | Yes (2) | Yes (6) | - |
| LEVY1 | Yes | Yes | Yes | Yes | See paper – situation different |
| LEVY2 | Yes (1) – only as e-cigs | Yes | No | No | This paper is concerned with replacing cigarettes with e-cigarettes. There is no initiation of cigarettes or dual use |
| UM | Yes (1) – only as smoking | Yes (1) – only from smoking | No | No | - |
|  |  |  |  |  |  |

Notes: The six tobacco industry sponsored models are shown first. Numbers in parentheses are counts of allowed transitions. Note that for models which only allow for two initiation probabilities, dual use may still be achieved by initiating one of the two products and then switching to dual use. Similarly quitting from dual use, or re-initiation to dual use can occur in two transitions.

^a^ From never tobacco user to current user of one or both products.

^b^ From current user to one or both products to former (or never) user of both products.

^c^ From former users of one or both products and current user of neither to current user of one or both products.

^d^ From one current use group to another.

^e^ Note that the description of the transitions in Figure 1 of the source paper (Vugrin et al., 2015) describes the terms quitting, re-initiation and switching differently, according to what happens to use of a single product. For example, individuals transitioning from current use of both products to current use of only one would be regarded as quitters by FDA, but as switchers from dual use to single product use in Table 2. Also note that Figure 1 illustrates 27 transitions, but only names 16 (with letters from a to p) and it is unclear whether all 27 are used in the modelling.

^f^ Analyses were based on input data that excluded experimenters and short-term quitters, so that cessation followed by relapse occurring more than twice was highly unlikely. By restricting the number of times transitions could occur, RJR could allow TTPs to depend on previous transitions.

^g^ ALCS2 has 27 possible transitions, but not all were used in the alternative scenario described in Table 3.

#### Supplementary Table 5: Fuller description of the methodology for estimating the population health impact from the tobacco histories developed

This supplementary table gives detailed information on how death rates and/or relative risks are derived from the tobacco histories developed using the tobacco transition probabilities. In the following, r refers to rates and RR to relative risks (compared to the non-exposed). Subscripts attached to them, where applicable, describe various tobacco groups, including n = non-exposed cs = current exclusive smokers, cm = current exclusive MRTP users, and cd = current dual users, fs = former smokers, ysc = years smoked cigarettes, ysm = years smoked MRTP, yqc = years quit cigarettes and yqm = years quit MRTP. F and G refer to the increase in risk for a current MRTP user or a current dual user expressed relative to the increase in risk for a current cigarette smoker. H refers to the quitting half-life, the time it takes following quitting for the excess relative risk (ER = RR–1) to be halved.

Seven of the models (ALCS1, ALCS2, BAT, JTI, RJR, FDA, UM) remove deaths during follow-up, and can thus readily calculate and compare the number of deaths occurring during follow-up (or periods of it) in the Null and Alternative Scenarios. They can also calculate loss of life from the ages at which these deaths occur. For these models I do not comment further on the derivation of these indices of population health impact from the estimated death rates, making comments only for the other three models (PMI, LEVY1, LEVY2) which do not remove deaths.

##### PMI

Using the baseline distribution of smoking habits (never, current, former by time quit), an assumed age at starting to smoke for current and former smokers, and the transition probabilities in the Null and Alternative Scenarios, PMI develop a full history of smoking and MRTP use over time. RRs compared to never use for each individual are then estimated at each year of follow-up using an extended negative exponential model. Apart from knowing the tobacco status of each individual at each year of follow-up, the method requires estimates by age, obtained from meta-analyses (Lee et al., 2017), of the RR for a current cigarette smoker, and of H.

The method of estimating the RR for an individual by age for each disease considered depends on first calculating what is termed an equivalent dose (ED) at each age, and then multiplying this by the ER for a continuing cigarette smoker of that age. ED starts at 0, as no one uses tobacco at birth. As a switch occurs, ED gradually increases towards the relative exposure (RE) value for the product switched to, where RE is 1 for a current cigarette smoker, F for a current MRTP user, G for a current dual user and 0 for a quitter. Then, as RE increases (or decreases) following a further switch, ED gradually increases (or decreases) towards the new RE value. Formally, if at age a, RE(a) is the relative exposure, and H(a) is the half-life, one first calculates the negative exponential factor for a single year as

$N(a)= exp (ln(2)/H(a))$

and then, with ED (1) taken as 0, calculates subsequent ED values by

$ED\left( a \right)=N\left( a \right)ED\left( a-1 \right)+\left( 1 -N\left( a \right) \right) RE(a)$

For a given sex and age the mean RR for all individuals is then estimated as $\overline{RR .}\mathrm{Then}P=(\bar{RR}-1)/\bar{RR}$ is taken as the proportion of deaths attributable to tobacco. and the total number of deaths from that disease in the national population is then multiplied by P to give the numbers of deaths attributable to tobacco. Years of life lost before age 75 are then estimated from these numbers using the midpoints of the five year age groups, e.g. 75-42.5 = 32.5 for a death at age 40-44.

##### JTI

This is quite similar to the method used by PMI, though the RRs are for all cause mortality and derived from CPSII. As for PMI it is assumed that, after a smoker quits, ER decays exponentially from its original value ER(0) to its value after time t = ER(0) exp (–kt) where k is the decay slope, or equivalently ln(2)/k is the half-life. Thus, ER(t) approaches 0 asymptotically. Generalizing this asymptote to any equilibrium value ER_eq_ leads to

$ER(t)=ER\left( 0 \right)\exp(-kt)+{ER}_{eq}\left[ 1-exp(-kt) \right]$

$=ER\left( 0 \right)+\left[ {ER}_{eq}-ER(0) \right]\left[ 1-\exp(-kt) \right]$

The authors note that the parameters k and ER_eq_ are not necessarily constant, the annual update making it straightforward to vary both k and ER_eq_ over time. They incorporated the slowing of ER decay rates k with age in this way, and also changed ER_eq_ with product use changes. Death rates were estimated by multiplying the RR (i.e. ER + 1) by the never smoker death rate.

##### BAT

Whereas PMI and JTI follow individuals though time, BAT follows groups. Current smoker RRs were taken as 1.00 for ages <35 and then as 1.81 for males and 1.66 for females, based on published UK data. Former smoker RRs were also taken as 1.00 for ages <35 and then as values declining from the current smoker values, estimated for grouped years quit using an H of 9.08 years, fitted to known former smoker RR data. Given also age and sex dependent never smoker mortality rates, the probability of death for groups divided by sex, age and smoking habits could then be calculated straightforwardly in the Null Scenario.

In the Alternative Scenario ERs were calculated for each sex as follows:

| MRTP user who had never smoked | ER_1_ = CE x F |
| --- | --- |
| Current MRTP user with a history of smoking | ER_2_  = CE x (F+ (1 – F) exp^–A^) |
| Current dual user | ER_3_ = Maximum of CE and ER_1_ |
| Former MRTP user who has never smoked | ER_4_  = CE x F x exp^–B^ |
| Former MRTP user with a history of smoking | ER_5_  = Maximum of ER_2_ and ER_4_ |
| Former dual user | ER_6_  = Maximum of CE x exp^–A^ ER_4_ |

In the above current CE is the current smoker ER, F is the F-factor, A is time quit smoking multiplied by ln(2)/H and B is time quit MRTP multiplied by ln(2)/H.

##### RJR

RJR also follow groups. In their first paper (Bachand and Sulsky, 2013) which describes the model and uses the population health impact of introducing snus as an illustrative example, they fitted Poisson models to data from the Kaiser-Permanente cohort study which provides mortality rates for men by age, years of smoking and years of quitting. In Appendix A, they presented detailed formulae showing how rates were derived for nine groups, and stated that the modelled mortality estimates were validated using actual population life tables. Simplifying the formulae ad denoting years smoked as ysc and years quit as yqc we have:

| 1. | Mortality rate for never smokers |  | r_1_ = exp (β_0_ + β_1_ age + β_2_ age^2^) |
| --- | --- | --- | --- |
| 2. | RR for current smokers |  | RR_2_ = exp (β_3_ysc + β_4_ age×ysc) |
| 3. | RR for former smokers |  | RR_3_ = RR_2_* exp (β_5_yqc + β_6_ age×yqc) |
| For MRTP users we first define | |  |  |
|  |  | A= | (RR for current MRTP – 1) /  (RR for current smoking – 1) |
|  |  | B= | (RR for former MRTP – 1) /  (RR for former smoking – 1) |
| We then have | |  |  |
| 4. | RR for current MRTP users |  | RR_4_ = A* (RR_2_–1) + 1 |
| 5. | RR for former MRTP users |  | RR_5_ = B* (RR_3_–1) + 1 |
| 6. | RR for smokers switching to MRTP |  | RR_6_ = RR_2_*RR_4_* ((1–A) RR_3_+A) |
| 7. | RR for MRTP users switching to smoking |  | RR_7_ = RR_2_*RR_4_ |
| 8. | RR for those who switch to MRTP then quit |  | RR_8_ = RR_2_*RR_5_* ((1–A)RR_3_+A) |
| 9. | RR for MRTP users who switch to smoking then quit |  | RR_9_ = RR_3_*RR_4_ |

Rates for groups 2 to 9 were estimated by multiplying RR_2_ to RR_9_ by r1.

##### ALCS1

ALCS1 also follow groups, and like RJR use data from the Kaiser-Permanente study on overall mortality by age, gender, duration of smoking and duration of quitting. Poisson models were used to estimate mortality for never, current and former smokers. The three separate models used were as follows:

| Rate in never users of tobacco | rN = exp (β_0_ + β_1_ age + β_2_ age^2^) |
| --- | --- |
| Rate in current cigarette smokers | rcs = exp (β_0_ + β_1_ age + β_2_ age^2^ + β_3_ys + β_4_ age*ys) |
| Rate in former cigarette smokers | rfs = exp (β_0_ + β_1_ age + β_2_ age^2^ + β_3_ys + β_4_yq + β_5_ age*ys + β_6_ age*yq) |

Mortality rates of exclusive current MRTP users and exclusive former MRTP users were estimated from the corresponding estimate for current exclusive smokers and current exclusive former smokers based on the ERR ratio = (RR (MRTP) – 1) / (RR (smoking) – 1).

Thus, if r_cm_ is the rate in current MRTP users we have

E = (r_ms_/r_n_ – 1) / (r_c_/r_n_ – 1)

or E = (r_m_ – r_N_) / (r_c_ – R_N_)

or r_m_ = E(r_c_ – r_N_) + r_N_

Detailed information on the derivation of the accumulated risks associated with spending time in each state based on a cohort member’s specific pathway can be found in the Supplemental File 1 submitted with Muhammad-Kah et al. (2019).

##### ALCS2

ALCS2 follows individuals rather than groups, but the estimation of the mortality rates is as in ALCS1.

ALCS 2 follows individuals rather than groups and also uses data from the Kaiser-Permanente study on overall mortality by age, gender, duration of smoking and duration of quitting. A Gompertz hazard function ${(h}_{age})$ that is dependent on the agent’s attained age, years smoked (YSM), years since cessation from cigarettes (YQSM), years of MRTP use (YM) and years since cessation from MRTP use (YQM), was used to estimate mortality. These five factors must be set in order to determine the probability of mortality for any given age and tobacco use history.

Equation 1: *h*(age, YSM, YQSM, YM, YQM)

=${(e}^{{\theta_{HMD.2000}*\alpha}_{HMD.2000}+ \beta_{HMD.2000} * age})*$ $\left( e^{\gamma_{KP,NT\&CS} * YSM + \delta_{KP,NT\&CS} * age*YSM+ \eta_{KP,CS\&FS} * YQSM} \right)$ *

$\left( 1+ \boldsymbol{\kappa}*\left( e^{\gamma_{KP,NT\&CS} * YM + \delta_{KP,NT\&CS} * age * YM+\eta_{KP,CS\&FS} * YQM}-1 \right) \right)$

Where the mortality rates for MRTP users can be modeled by setting the ERR ratio value = κ as defined in ALCS1 and the coefficients are derived from regression models e.g. $\gamma_{KP,NT\&CS}$

The mortality probability of an agent is calculated from the hazard rates using the equation below:

$P\left( Mortality \right)=1-e^{[-h\left( age, YSM, YQSM, YM, YQM \right)]}$

##### FDA

FDA derive RR estimates by gender, age, smoking status and age quit from the National Health Interviews. In their first paper (Vugrin et al., 2015) they estimate rates for ever users of the MRTP as follows:

| 1. | Never used either product | RR_1_ = 1 |
| --- | --- | --- |
| 2. | Current exclusive smoker | RR_2_ = RR_cs_ |
| 3. | Former exclusive smoker | RR_3_ = RR_fs_ |
| 4. | Current exclusive MRTP user | RR_4_ = 1 + F(RR_2_ – 1) |
| 5. | Current dual user | RR_5_ = Maximum of RR_2_ and RR_4_ |
| 6. | Former smoker, current MRTP user | RR_6_ = RR_3_ + F (RR_2_ – RR_3_) |
| 7. | Former exclusive MRTP user | RR_7_ = 1 + F(RR_3_ – 1) |
| 8. | Current smoker, former MRTP user | RR_8_ = RR_2_ |
| 9. | Former smoker, former MRTP user | RR9 = Maximum of RR_3_ and RR_7_ |

In their second paper (Apelberg et al., 2018), which considers chewing tobacco or snuff as the MRTP, some RRs are modified to use published information on the RR for use of these products. RR3_4_ is replaced by the actual RR for current exclusive use of chewing tobacco or snuff, while RR_5_ is taken as 8% higher than RR_3_ as long as (a) 1.08 RR_3_ is not greater than RR_2_, when it is set to RR_2_ and (b) 1.08 RR_3_ is not less than RR_4_, when it is set to RR_4_. Also RR_7_ is set as 1.

Given the RRs, and the death rates for never users of either product, the ratio for the seven groups of tobacco users can then be derived.

##### LEVY1

This model assumed that initiation of smoking and MRTP occurred up to age 25, with individuals progressing from trial use to a single state of exclusive cigarette, exclusive MRTP, dual use or no use. After age 25, cessation of exclusive cigarette smoking, exclusive MRTP use and dual use was assumed to occur at the rate of smoking cessation under the Null Scenario. Age and gender dependent RRs for smoking come from CPSI and CPSII, while for exclusive MRTP or dual users, RRs were taken as 1 + F (RR_cs_ – 1) or 1 + G (RR_cs_ – 1). Based on the distribution of smoking habits and the RRs, for the Null Scenario, the number of smoking-attributable deaths was calculated for current and former smokers for each age in the 1997 cohort as the product of excess risks and the corresponding population multiplied by the prevalence rate. The number of life-years lost at each age was estimated as the product of the number of premature deaths and the expected years of life remaining for a never smoker. For each MRTP Scenario, smoking-attributable deaths and years of life lost were calculated for current and former exclusive smokers, exclusive MRTP users and dual users at each age and then summed. The population health impact was then estimated by the differences in smoking-attributable deaths and years of life lost in the two Scenarios.

##### LEVY2

In this model, the Alternative Scenarios tested were ones in which cigarettes were largely replaced by the MRTP over a 10 year period. As for LEVY1, age and gender dependent RRs by smoking came from CPSI and CPSII. However, the RRs used for ever users of MRTP were somewhat different. Thus, where RR_sm_ is the risk in current smokers and RR_fs_ is the risk on former smokers.

| 1. | Current MRTP users who never smoked cigarettes |  | RR_1_ = (1 – F) + F*RR_cs_ |
| --- | --- | --- | --- |
| 2. | Current MRTP users who switched from cigarettes before age 40 |  | RR_2_ = RR_1_ |
| 3. | Current MRTP users who switched from cigarettes after age 40 |  | RR_3_ = (1–F) RR_fs_+ F*RR_sm_ |
| 4. | Former MRTP users who never smoked cigarettes |  | RR_4_ = (1–F) + F*RR_fs_ |
| 5. | Former MRTP users who switched from cigarettes before age 40 |  | RR_5_ = RR_4_ |
| 6. | Former MRTP users who switched from cigarettes after age 40 |  | RR_6_ = (1–F) RR_fs_ + F*RR_4_ |

Given this, the estimation of population health impact was as described in LEVY1

##### UM

UM only considers risk relating to smoking, e-cigarette only having a role by inducing smoking initiation and cessation. At each year of follow-up, the distribution of survivors in each smoking group, combined with overall data on age-specific death rates and estimates from CPS II of relative mortality rates by gender, age and smoking status are used to determine the number of survivors during in the year.

#### References

Apelberg, B.J., et al., 2018. Potential public health effects of reducing nicotine levels in cigarettes in the United States. N. Engl. J. Med. 378, 18, 1725-1733. DOI:10.1056/NEJMsr1714617.

Bachand, A.M., Sulsky, S.I., 2013. A dynamic population model for estimating all-cause mortality due to lifetime exposure history. Regul. Toxicol. Pharmacol. 67, 2, 246-251. DOI:10.1016/j.yrtph.2013.08.003.

Bachand, A.M., Sulsky, S.I., Curtin, G.M., 2018. Assessing the likelihood and magnitude of a population health benefit following the market introduction of a modified-risk tobacco product: enhancements to the dynamic population modeler, DPM(+1). Risk Anal. 38, 1, 151-162. DOI:10.1111/risa.12819.

Black, R., Murillo, J., Pithawalla, Y., 2018. Population modeling. A practical approach to evaluate the net benefit of a new tobacco product. Tobacco Computational Modeling Meeting, NYU College of Global Public Health. Altria Client Services (ALCS)**.**

Djurdjevic, S., Lee, P.N., Weitkunat, R., Sponsiello-Wang, Z., Ludicke, F., Baker, G., 2018a. Modeling the population health impact of introducing a modified risk tobacco product into the U.S. market. Healthcare (Basel). 6, 2, 47; doi: 10.3390/healthcare6020047. DOI:10.3390/healthcare6020047.

Djurdjevic, S., Sponsiello-Wang, Z., Lee, P.N., Fry, J.S., Weitkunat, R., Ludicke, F., Baker, G., 2018b. Modeling the impact of changes in tobacco use on individual disease risks. Regul. Toxicol. Pharmacol. 97, 88-97. DOI:10.1016/j.yrtph.2018.06.001.

Fisher, M.T., Tan-Torres, S.M., Gaworski, C.L., Black, R.A., Sarkar, M.A., 2019. Smokeless tobacco mortality risks: an analysis of two contemporary nationally representative longitudinal mortality studies. Harm Reduct. J. 16, 1, 27. DOI:10.1186/s12954-019-0294-6.

Hill, A., Camacho, O.M., 2017. A system dynamics modelling approach to assess the impact of launching a new nicotine product on population health outcomes. Regul. Toxicol. Pharmacol. 86, 265-278. DOI:10.1016/j.yrtph.2017.03.012.

Lee, P.N., Fry, J.S., Hamling, J.F., Sponsiello-Wang, Z., Baker, G., Weitkunat, R., 2017. Estimating the effect of differing assumptions on the population health impact of introducing a Reduced Risk Tobacco Product in the USA. Regul. Toxicol. Pharmacol. 88, 192-213. DOI:10.1016/j.yrtph.2017.06.009.

Levy, D.T., et al., 2018. Potential deaths averted in USA by replacing cigarettes with e-cigarettes. Tob. Control. 27, 1, 18-25. DOI:10.1136/tobaccocontrol-2017-053759.

Levy, D.T., et al., 2017. The application of a decision-theoretic model to estimate the public health impact of vaporized nicotine product initiation in the United States. Nicotine Tob. Res. 19, 2, 149-159. DOI:10.1093/ntr/ntw158.

Muhammad-Kah, R.S., Pithawalla, Y.B., Boone, E.L., Wei, L., Jones, M.A., Black, R.A., Bryan, T.M., Sarkar, M.A., 2019. A computational model for assessing the population health impact of introducing a modified risk claim on an existing smokeless tobacco product. Int. J. Environ. Res. Public Health. 16, 7. DOI:10.3390/ijerph16071264.

Nutt, D.J., et al., 2014. Estimating the harms of nicotine-containing products using the MCDA approach. Eur. Addict. Res. 20, 5, 218-225. DOI:10.1159/000360220.

Poland, B., Larroque, S., 2018. Probabilistic analysis of the effects of a modified risk tobacco product on population health [abstract POS4-134]. Society for Research on Nicotine & Tobacco. Annual Meeting, Baltimore, MD. February 21-24, 2018. Available: <https://www.jt-science.com/sites/default/files/2018-p1-jti.pdf>.

Poland, B., Teischinger, F., 2017. Population modeling of modified risk tobacco products accounting for smoking reduction and gradual transitions of relative risk. Nicotine Tob. Res. 19, 11, 1277-1283. DOI:10.1093/ntr/ntx070.

Tam, J., Day, H.R., Rostron, B.L., Apelberg, B.J., 2015. A systematic review of transitions between cigarette and smokeless tobacco product use in the United States. BMC Public Health. 15, 258. DOI:10.1186/s12889-015-1594-8.

Vugrin, E.D., Rostron, B.L., Verzi, S.J., Brodsky, N.S., Brown, T.J., Choiniere, C.J., Coleman, B.N., Paredes, A., Apelberg, B.J., 2015. Modeling the potential effects of new tobacco products and policies: a dynamic population model for multiple product use and harm. PLoS One. 10, 3, e0121008. DOI:10.1371/journal.pone.0121008.

Warner, K.E., Mendez, D., 2019. E-cigarettes: Comparing the possible risks of increasing smoking initiation with the potential benefits of increasing smoking cessation. Nicotine Tob. Res. 21, 1, 41-47. DOI:10.1093/ntr/nty062.

Weitkunat, R., Lee, P.N., Baker, G., Sponsiello-Wang, Z., González-Zuloeta Ladd, A.M., Lüdicke, F., 2015. A novel approach to assess the population health impact of introducing a modified risk tobacco product. Regul. Toxicol. Pharmacol. 72, 87-93. DOI:10.1016/j.yrtph.2015.03.011.
