# Supplementary material for: Estimating the Population Health Impact of Recently Introduced Modified Risk Tobacco Products: A Comparison of Different Approaches
Source: Nicotine Tob Res. 2020 Jun 4;23(3):426–37. doi: 10.1093/ntr/ntaa102 (PMC7885777; doi:10.1093/ntr/ntaa102)
Supplement: ntaa102_suppl_Supplementary_File_2 [file ntaa102_suppl_supplementary_file_2.docx]

“Estimating the population health impact of recently introduced modified risk tobacco products: a comparison of different approaches”

By Peter N Lee et al.

Supplementary File 2 (Figures)

Date: March 2020

Figure 1 shows the transitions that are possible in the Null Scenario, where the MRTP is not introduced.

Figure 2 shows the transitions that are possible in the Alternative Scenario for the PMI model.

See also the text in the main paper under “tobacco transition probabilities”, and Supplementary Tables 3 and 4.

#### Figure 1 : Transitions in the Null Scenario

|  | Smoking group at time t+1 | | |
| --- | --- | --- | --- |
| Smoking group time t | Never smoked | Current smoker | Former smoker |
|  |  |  |  |
| Never smoked |  | | |
|  |  | | |
|  |  | | |
| Current smoker |  | | |
|  |  | | |
|  |  | | |
| Former smoker |  | | |
|  |  | | |

The lines in black represent no change, while the lines in red, blue and orange correspond, respectively, to initiation, quitting and re-initiation.

Transitions from current or former to never smoker are not possible.

Transitions from never smoker to former smoker typically take two or more time periods.

#### Figure 2 : Transitions in the Alternative Scenario (PMI model)

|  | Product group at time t+1 | | | | |
| --- | --- | --- | --- | --- | --- |
| Smoking group at time t | Neither either product | Current cigarette only | Current MRTP only | Current dual use | Former product use |
|  |  |  |  |  |  |
| Never either |  | | | | |
| product |  | | | | |
|  |  | | | | |
|  |  | | | | |
|  |  |  |  |  |  |
| Current |  | | | | |
| cigarette only |  | | | | |
|  |  | | | | |
|  |  | | | | |
|  |  |  |  |  |  |
| Current MRTP |  | | | | |
| only |  | | | | |
|  |  | | | | |
|  |  | | | | |
|  |  |  |  |  |  |
| Current dual |  | | | | |
| use only |  | | | | |
|  |  | | | | |
|  |  | | | | |
|  |  |  |  |  |  |
| Former |  | | | | |
| product use |  | | | | |
|  |  | | | | |
|  |  | | | | |

The lines in black represent no change, while the lines in red, blue, orange and green correspond, respectively, to initiation, quitting, re-initiation and switching.

Transitions from current or former user to never user are not possible.

Transitions from never user to former user typically take two or more time periods.
